# The TCF7L2/miR-206/Cofilin1 axis promotes the metastasis of bladder cancer cells by regulating the formation of invadopodia: TCF7L2/miR-206/Cofilin1 regulates invadopodia

**DOI:** 10.3724/abbs.2025114

**Published:** 2025-08-06

**Authors:** Yuzhen Jie, Yinggui Yang, Chengyan Guo, Qinghui Wu, Zhewen Ou, Weifu Wang, Ning Xu, Wei Peng, Yingguang Wu, Jiangfan Peng, Shengchao Ma, Shufang Zhang, Fei Wang

**Affiliations:** 1 Department of Urology Affiliated Hainan Hospital of Hainan Medical University Hainan General Hospital Hainan Provincial Clinical Medical Center Haikou 570311 China; 2 School of Clinical Medicine Hainan Medical University Haikou 571199 China; 3 Department of Urology Shenzhen Hospital of Southern Medical University Shenzhen 518110 China; 4 NHC Key Laboratory of Metabolic Cardiovascular Diseases Research Ningxia Medical University Yinchuan 750004 China; 5 Central Laboratory Haikou Affiliated Hospital of Central South University Xiangya School of Medicine and Haikou City Key Laboratory of Clinical Medicine Haikou 570203 China

**Keywords:** bladder cancer, metastasis, Cofilin1, invadopodia formation

## Abstract

Bladder cancer (BCa) is one of the most common malignant tumors of the urinary system, but its pathogenesis is still unclear. T1G3 BCa is particularly invasive and relapses readily after treatment, with progression to invasive cancer or distant metastasis. Therefore, identification of the molecular mechanism by which it invades and metastasizes to guide treatment and predict patient prognosis is needed. Cofilin1 plays an important role in regulating gene expression and the invasiveness of tumors. In this study, we show that Cofilin1 is highly expressed in BCa and lymph nodes with metastasis, which is positively related to the grade of BCa, and is significantly related to clinicopathological parameters and cancer-specific survival. Phenotypic analysis reveals that *Cofilin1* knockout inhibits the proliferation and migration of BCa cells, whereas Cofilin1 overexpression promotes the opposite phenotype. Cofilin1 binds to cortactin, thereby reducing the expression of F-actin and promoting the formation of invadopodia in BCa cells. Further experiments reveal that TCF7L2 can bind to the promoter of *Cofilin1* and transactivate it, promoting a malignant phenotype. TCF7L2 may also reverse the inhibitory effect of miR-206 on the binding of Cofilin1 and cortactin and promote the metastasis of BCa by inhibiting the transcription maturation of miR-206. This study confirms that *Cofilin1* is an oncogene in T1G3 BCa, and the TCF7L2/miR-206/Cofilin1 signaling pathway plays an important role in the formation of invadopodia in BCa.

## Introduction

Bladder cancer (BCa) is one of the most common urological malignancies and is highly invasive with a poor prognosis [1]. In 2020, there were 573,000 new cases of BCa and 213,000 deaths from the disease worldwide [2]. Approximately 30% of patients with BCa have invasive disease at initial presentation. Moreover, even with radical surgery, the 5-year overall survival rate for these patients is only 50% [3]. Given the highly invasive nature of BCa and the lack of effective treatments, it is important to elucidate the mechanisms by which BCa invades and metastasizes at the molecular level and identify early molecular markers of BCa with high potential for invasion to guide the choice of treatment for T1G3 BCa and improve the survival rate. Transcription factor 7-like protein 2 (TCF7L2) is a key regulator of the Wnt signaling pathway and has been shown to be important in the development and progression of a variety of malignant tumors [ 4, 5] . MiR-206 is a microRNA that is aberrantly expressed in a variety of tumors and is involved in the regulation of their invasion and metastasis [6]. Cofilin1 belongs to the actin depolymerization factor family and is an actin-binding protein that regulates cytoskeletal remodeling and is closely related to cell migration and invasion [7]. Previous studies have demonstrated that Cofilin1 is significantly upregulated in melanoma and ovarian cancer tissues [ 8, 9] and that the expression of activated forms of Cofilin1 is markedly elevated in some cancers, including those of the breast, lung, and prostate [ 10– 12] , whereas the expression of phosphorylated inactivated Cofilin1 is markedly reduced. Activated Cofilin1 induces the formation of invadopodia in tumor cells and enhances their potential for proliferation and invasion. Conversely, silencing of the *Cofilin1* gene results in a significant decrease in activated free Cofilin1, which leads to a significant reduction in the generation of invadopodia and disordered maturation in tumor cells, thereby inducing a marked decrease in proliferation and invasiveness. Although Cofilin1 has been shown to be closely related to the development of BCa [ 13, 14] , relevant studies have focused on confirming their correlation without exploring the regulatory function or the potential molecular mechanism by which Cofilin1 promotes the invasion and metastasis of BCa.

Therefore, the present study aimed to explore the mechanism by which the TCF7L2/miR-206/Cofilin1 signaling axis induces the formation of invadopodia and the metastasis of BCa. Our initial internal and external experiments detected the expressions of TCF7L2, miR-206, and Cofilin1 in BCa tissues and cells. Next, we explored the expressions of TCF7L2 and miR-206 by transfection techniques and further observed their effects on the invasiveness and formation of invadopodia in T24 BCa cells. Finally, we confirmed the role of the TCF7L2/miR-206/Cofilin1 signaling axis in the metastasis of BCa in an animal model.

## Materials and Methods

### Animal models

Thirty-six specific pathogen-free female BALB/C-NU mice (aged 4–6 weeks) were obtained from Guangzhou East Provincial Medical Experimental Animal Center (certificate number 44007200028760) (Guangzhou, China). The mice were kept at a controlled temperature of 22 ± 3°C with the humidity controlled at 50% ± 20% and a 12/12-h light/dark cycle. Food and water were freely available. Specific pathogen-free-grade rat feed was supplied by the Guangdong Medical Experimental Animal Center (certificate number 0082700). All the animals were kept in the experimental animal room for at least 3 d to acclimatize to the environment.

### Cell culture and transfection

A normal human uroepithelial (SV-HUC-1) cell line and human BCa (BIU-87, EJ, T24, and RT4) cell lines were obtained from the Cell Bank at the Chinese Academy of Sciences (Shanghai, China). All the cells were routinely cultured in an incubator at 37°C with 5% CO _2_ in RPMI-1640 medium (Hyclone, Carlsbad, USA) containing 10% fetal bovine serum (FBS) and 1% double antibiotics according to standard procedures. The cells were passaged when they reached 80% confluence. Next, 2 × 10 ^5^ cells were inoculated into 24-well plates and cultured for 24 h. Plasmids or RNA were then differentially transfected using Lipofectamine 2000 reagent (Invitrogen, Carlsbad, USA) according to the manufacturer’s instructions. At 24 h post-transfection, the cells were harvested for further experiments. The short hairpin RNA (shRNA) and plasmid sequences of *Cofilin1*, *Cortactin*, *TCF7L2* and *miR-206* are listed in Supplementary Table S1.

### Patients and clinical samples

We selected 10 pairs of cancerous and paracancerous tissues and 2 cases each of metastatic BCa and lymph node tissues from patients who underwent radical cystectomy at Hainan General Hospital between 2018 and 2022. The patients’ background characteristics are shown in Supplementary Table S2. The study was approved by the Hainan General Hospital institutional ethics committee (approval number 201727). All patients who provided tissue samples provided written informed consent. Cancerous and paired paracancerous tissues were collected after radical BCa surgery, and their histological characteristics were confirmed pathologically.

### Western blot analysis

The clinical tissue homogenates were collected from each group and added to lysis solution until they were fully lysed. The lysed cell samples were transferred to 1.5-mL centrifuge tubes and centrifuged at 12,000 *g* for 10 min. Next, the supernatant was removed, and its protein concentration was determined using the bicinchoninic acid reagent (Beyotime, Shanghai, China) according to the manufacturer’s instructions. The protein lysates were separated by 8% SDS-PAGE and transferred to a 0.22-μm polyvinylidene fluoride membrane (Millipore, Darmstadt, Germany). After the membranes were blocked with 5% skim milk for 2 h at room temperature, they were incubated with the corresponding specific primary antibodies overnight at 4°C, and then incubated 2 h at room temperature with horseradish peroxidase-labelled goat anti-mouse IgG or goat anti-rabbit IgG, which were added at a dilution ratio of 1:5000 ( Supplementary Table S3). The signals were visualized using an enhanced chemiluminescence reagent (Thermo Fisher Scientific, Waltham, USA), and the gray values of the immunoblots were determined using Image Lab 5.0 (Bio-Rad, Hercules, USA).

### Quantitative real-time PCR

Total RNA was extracted from cancerous and paracancerous tissues using TRIzol reagent (Invitrogen, Carlsbad, USA) according to the manufacturer’s instructions. The RNA quality was determined via formaldehyde denaturing agarose gel electrophoresis, and cDNA was synthesized using HiScript II Q RT SuperMix for qPCR (Vazyme, Nanjing, China) according to the manufacturer’s instructions. Commercially available *Cofilin1* and the internal reference *18S rRNA* Taqman probe (Applied Biosystems, Waltham, USA) were used for qRT-PCR. The sequences of primers used in the experiments are shown in Supplementary Table S4. The PCR amplification procedure included predenaturation for 30 s at 95°C, denaturation for 5 s at 95°C, annealing for 34 s at 60°C, and extension for 30 s at 72°C for 40 cycles. The same system was set up with a blank control and an internal reference ( *18S rRNA*/ *β-actin*) control for amplification. On the basis of the amplification curve, the content of *Cofilin1* mRNA was analyzed and normalized according to the calculation formula (2 ^–ΔΔCt^).

### Hematoxylin-eosin staining

The bladder cancer tissue specimens with different pathological grades were fixed with formaldehyde, dehydrated with ethanol, immersed in xylene for 2 min, and embedded in paraffin. Then they were sliced, dewaxed, stained with hematoxylin-eosin, sealed, and observed and photographed under a microscope (SZ61; Olympus, Tokyo, Japan).

### Immunohistochemistry (IHC)

The tissue microarrays were fixed with 4% paraformaldehyde and then subjected to routine immunohistochemistry with anti-Cofilin1 antibodies. The expression levels of Cofilin1 were determined *in situ* in metastatic cancerous and paracancerous samples and evaluated after staining. The correlations of the Cofilin1 expression level with the clinicopathological features and prognosis of patients with BCa were analyzed.

### *In vivo* xenograft tumor model

A total of 5 × 10 ^6^ control (Vector + shlacZ), Cofilin1-overexpressing or *Cofilin1*-knockdown RT4 or T24 cells were suspended at a 1:1 ratio of PBS:Matrigel (Corning, New York, USA) to 100 μL per injection and injected (25 g needle) subcutaneously into the backs of nude mice. Next, we observed the growth of the tumors, measured the longest and shortest diameters of each tumor every 3 days and calculated its volume via the formula V= *ab* ^2^/2 (where *a* represents the long diameter and *b* represents the short diameter). *In vivo* growth curves were then drawn for the cancer cells. Blood was collected at the end of the experiment; the serum was separated and frozen at –80°C for later use. The tumors were removed from the mice and photographed. The tumor blocks were fixed in 4% paraformaldehyde.

### Transwell migration and invasion assays

For the migration experiments, 1 × 10 ^5^ cells from the treatment group were collected, resuspended in 100 μL of serum-free medium, and added to the upper chamber of a transwell cell culture plate. For the invasion experiments, Matrigel was dissolved overnight at 4°C. Cells were then diluted with precooled serum-free medium at a volume ratio of 1:3, after which 1 × 10 ^5^ cells were counted. The cell mixture was added to the upper chamber of the transwell cell culture plate. The lower chambers were filled with medium containing 10% FBS. After incubation for 24 h, the cells were fixed in 4% paraformaldehyde for 15 min and stained with crystal violet for 10 min. The cells were observed under a microscope (Olympus) to determine whether they had passed through the wells and then photographed for counting.

### Colony formation assay

The cells from the various experimental groups were collected by trypsin digestion, and 1 mL of medium was added to form a single-cell suspension. The cells were then diluted to 1 × 10 ^3^ cells/mL and inoculated into 96-well plates, after which 50 μL ( *i*. *e*., 50 cells/well), 100 μL (100 cells/well), and 200 μL (200 cells/well) were sequentially removed for inoculation. The cells were shaken horizontally to distribute them evenly in the wells. After 7 days of culture, when the individual cells were observed to be growing into clusters of clones, 200 μL of crystal violet staining solution was added to cover the bottom of each well. The wells were then rinsed under tap water after 20 min and air-dried, after which the number of colonies was counted. The cells were analyzed, scanned, and photographed using an enzyme-linked speckle image autoanalyzer (Biotek, Winooski, USA).

### Cell adhesion assay

First, we added 50 μL of 30 mg/L fibronectin to each well of a 96-well plate, which was then placed on an ultraclean bench overnight for air-drying and to allow a fibronectin coating to form. Next, 20 μL of 3% bovine serum albumin in PBS was added to the plate, which was subsequently incubated at 37°C for 2 h. We added 200 μL (5 × 10 ^5^ cells/mL) of cells containing 10% FBS to each well, and the cells were incubated for 1–1.5 h. After incubation, the cells were washed twice with PBS to remove the cells that did not adhere. Six replicate wells were set up for each group, and photographs were obtained.

### Analysis of tumor gene databases

The Cancer Genome Atlas Program and cBioPortal for Cancer Genomics databases were used to detect *TCF7L2*, *miR-206*, and *Cofilin1* gene expressions in clinical BCa samples. The correlations of *TCF7L2*, *miR-206*, and *Cofilin1* expression levels with clinicopathological features were assessed using Pearson’s chi-square test. The expression levels of the *Cofilin1* and *TCF7L2* genes were determined for 33 cancers in the Tumor Immune Estimation Resource database. Survival analysis of key genes was performed using an online tool (GEPIA; http://GEPIA.cancer-pku.cn/) with the log-rank test and a Cox proportional risk regression model. Patients with BCa were categorized according to whether their gene expression was high or low on the basis of the median gene expression level. Survival curves were plotted for the key genes. A *P* value less than 0.05 was considered to indicate statistical significance. The diagnostic performance of each key gene was evaluated by plotting the ROC curve via the pROC software package in R and calculating the area under the ROC curve (AUC). The closer the AUC value is to 1, the more accurate the diagnosis.

### Immunofluorescence (IF) staining

First, 2000 T24 cells with good growth status were spread into a confocal Petri dish and then cultured for approximately 24 h until the cells were stretched and adherent to the wall of the dish. The cells were then fixed with 4% paraformaldehyde for 30 min, washed with cold PBS three times, permeabilized with 0.2% Triton X-100 for 5 min, and incubated at room temperature with anti-cortactin monoclonal antibody (Abcam, Cambridge, UK,) and then with secondary antibody at 4°C overnight. After elution, the number and morphology of the invadopodia were observed under a laser confocal microscope (ZEISS, Wetzlar, Germany). Five clear fields of view were randomly selected, and the percentage of invadopodia was calculated before the differences in the number of invadopodia in each group were compared.

### Live-cell imaging

The cells were digested and passaged approximately 36 h after transient transfection. Next, 2000 cells were spread into a confocal Petri dish and left to attach to the wall. Live-cell imaging was then performed via time-lapse photography to observe the formation of invadopodia.

### Transmission electron microscopy (TEM)

After the coverslips were placed on the 24-well plates, 200 μL of Matrigel was added. Transiently transfected cells were spread into 24-well plates and incubated for approximately 36 h until the cells were attached to the Matrigel. After the cells were fixed, the dried samples were prepared by soaking them in 100% isopentyl acetate, 1:1 benzene, and an epoxy propylene burner for 15 min and then in pure epoxy propylene compound at 45°C for 20 min, after which they were finally dried and vacuumed at the critical point. The dried samples were glued onto a metal platform via a conductive adhesive and placed in a vacuum evaporator to spray a 50–300 Å thick metal film, which was observed under a transmission electron microscope (Hitachi, Tokyo, Japan).

### Cell viability assay

The RT4 and T24 cell lines in the logarithmic growth phase were resuspended as a cell suspension and inoculated into 96-well plates at a density of 4000 cells/well. Each group of 3–5 wells was duplicated and cultured in a cell culture incubator. Starting from the second day after laying the plate, 10 μL of CCK-8 reagent (Beyotime) was added to the wells 2–4 h before termination of the culture without changing the medium. Four hours later, the 96-well plates were placed on an oscillator and shaken for 2–5 min. Finally, the absorbance at 450 nm was detected with a microplate reader, and the data were analyzed.

### Cell cycle assay

The RT4 and T24 sublines were seeded into 6-well plates at a density of 5 × 10 ^5^ cells/well. The medium was carefully aspirated no sooner than 12 h after inoculation. The cells were collected into tubes after two times wash with PBS. All the cells were treated with a cell cycle kit (KeyGEN, Nanjing, China) and then analyzed by flow cytometry.

### Apoptosis assay

The cells were collected after digestion with 2.5 g/L trypsin (without EDTA or phenol red) and washed three times with pre-cooled PBS. The negative control tubes and sample tubes were numbered according to the order of sampling. Next, 500 μL of 1× Annexin V conjugate was prepared at room temperature with protection from light; 100 μL of conjugate suspension, 5 μL of 7-AAD staining solution, and 5 μL of Annexin V/phycoerythrin staining solution were then added sequentially. The cells at the bottom of the tubes were gently stirred. When they were well mixed, they were incubated in the dark at 4°C for 15 min. After filtration using a 300-mesh filter sieve, the rate of apoptosis was detected in each group by flow cytometry.

### Dual luciferase assays

Luciferase reporter gene constructs containing wild-type or mutant *Cofilin1* 3′UTRs were obtained from GenePharma (Shanghai, China). The wild-type *Cofilin1* 3′UTR and the mutant *Cofilin1* 3′UTR were each cotransfected with the miR-206 mimic or miR-neg into HEK-293T cells using the Lipofectamine 3000 reagent. The cell lysates were collected 48 h after transfection and analyzed via a dual luciferase assay kit (Promega, Madison, USA).

### Ch-IP assay

Ch-IP assays were performed using the Magna Ch-IP G Assay kit (Thermo Fisher Scientific). The cells were crosslinked with 1% formaldehyde for 10 min at room temperature, and the reaction was quenched with glycine. Immunoprecipitation was performed via an antibody specific for TCF7L2 (Abcam), and DNA fragments of TCF7L2 bound to the miR-206 promoter region were enriched from sonicated cell lysates and subjected to PCR to amplify the TCF7L2/miR-206 binding sites.

### RNA immunoprecipitation assay

Magnetic beads (Thermo Fisher Scientific) were mixed with anti-AGO2 antibody (ab186773; Abcam) or normal rabbit IgG for 30 min. Cell lysates were immunoprecipitated with magnetic beads by gentle rotation at 4°C for 6 h. The magnetic beads were then washed five times. RNA was isolated from the protein-antibody-agarose complexes in accordance with the manufacturer′s protocol for qRT-PCR to detect RNA enrichment in the immunoprecipitated samples.

### RNA pull-down assay

The cells were harvested, lysed, and sonicated. A biotinylated TCF7L2 RNA probe and an Hsa-miR-206 probe were synthesized by RuiBo Biotechnology Co. Ltd. (Guangzhou, China). Briefly, 50 pmol of biotinylated TCF7L2/Hsa-miR-206 was incubated with streptavidin beads (Invitrogen) at 4°C overnight. Next, the cell lysates were incubated with streptavidin agarose beads (Invitrogen) for 1 h. After rinsing with washing buffer, the RNA-associated proteins were subject to silver staining. The samples to be tested after elution and purification were detected by qRT-PCR.

### Statistical analysis

All the quantitative data are expressed as the mean ± standard deviation (SD). Gene expression levels were compared between two groups via Student’s *t* test. One-way analysis of variance was used for comparisons among multiple samples. Two-by-two comparisons were made within groups, and the Pearson correlation coefficient was used to examine the correlation between two variables. All the statistical analyses were performed via GraphPad Prism version 10.0 (GraphPad Software Inc., La Jolla, USA) and SPSS version 22.0 (IBM Corp., Armonk, USA). *P*  < 0.05 was considered to indicate statistical significance.

## Results

### Cofilin 1 expression is upregulated in BCa tissues and cell lines and predominantly in the cytoplasm

**Figure 1:**
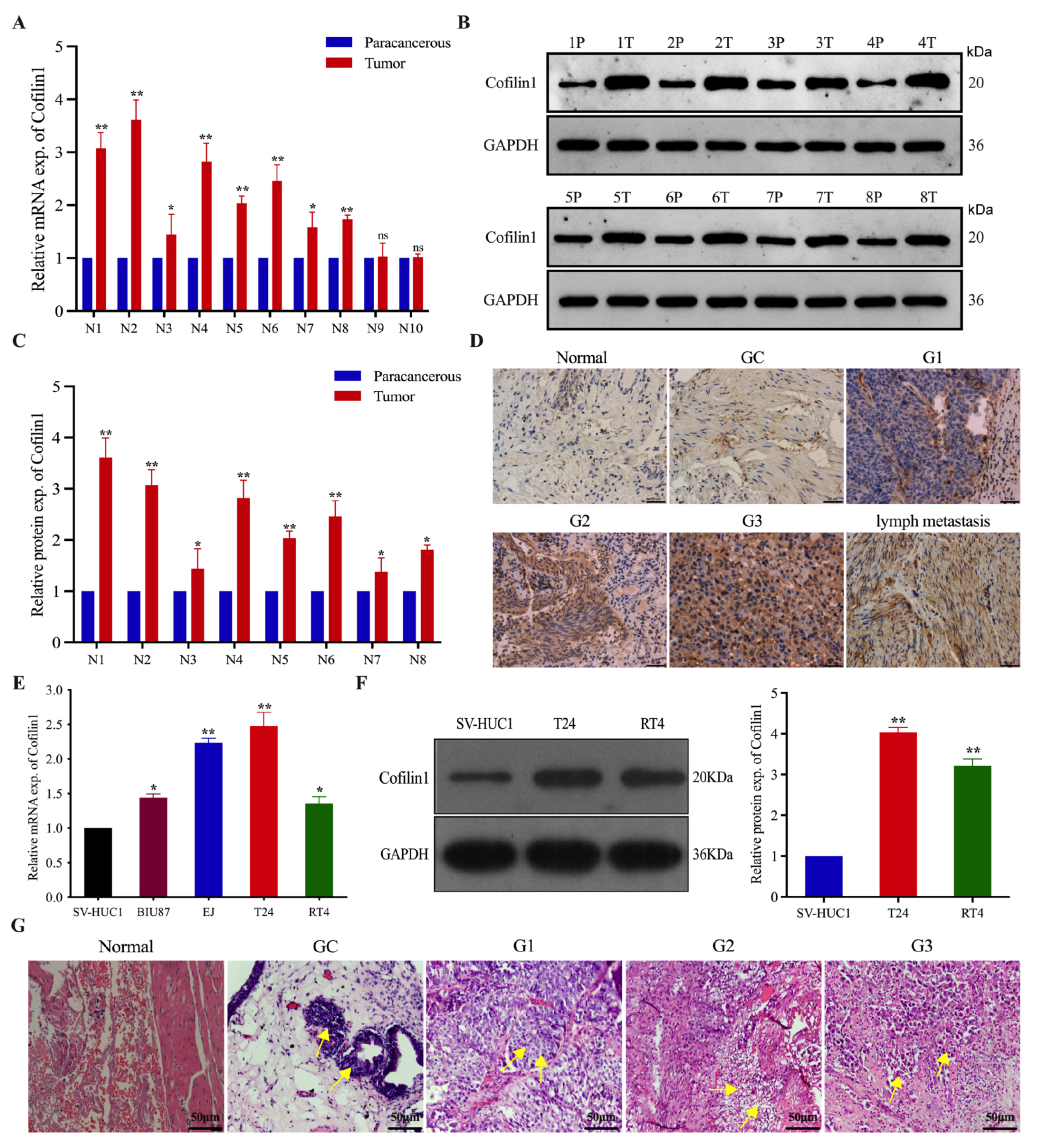
Figure 1 Cofilin1 expression is upregulated in BCa tissues and cell lines, mainly in the cytoplasm (A) The expression levels of Cofilin1 in BCa tissues and paracancerous tissues of 10 patients were detected by qRT-PCR, and β-actin was used as a reference control. Data are shown as the mean ± SD. n = 10. **P < 0.01. (B) Western blot analysis was used to detect Cofilin1 protein levels in BCa tissues of 8 cases with differential expression (n = 8). (C) Statistical graph of Cofilin1 protein expression detected by western blot analysis. (D) IHC detection of positive Cofilin1 expression in BCa tissues with different pathological grades, adenocystitis and paraneoplastic tissues Scale bar = 50 μm. n = 10. (E) qRT-PCR was performed to detect the transcript levels of Cofilin1 in different BCa cell lines (RT4, BIU-87, EJ, and T24) (n = 3). (F) Western blot analysis was used to determine the protein expression levels of Cofilin1 in SV-HUC-1, T24 and RT4 cell lines (n = 3). (G) Bladder cancer tissues were subject to HE staining, and the histological features of different stages were observed and compared by using a light microscope. Scale bar = 50 μm. n = 10. Data are expressed as the mean ± SD of three independent experiments. *P < 0.05, **P < 0.01, ***P < 0.001. Exp., expression.

First, we used qRT-PCR to detect *Cofilin1* mRNA expression levels in cancerous and paracancerous tissues from 10 patients. We found that *Cofilin1* expression was significantly higher in cancerous tissue than in paracancerous tissue in eight of the 10 patients ( Figure 1A). Next, by western blot analysis, we confirmed that Cofilin1 protein levels were significantly higher in cancerous tissues than in paracancerous tissues from these eight patients ( Figure 1B,C). Next, Cofilin1 expression was examined by immunohistochemistry in normal bladder mucosal tissues (8 cases), adenoid cystitis (8 cases), G1-grade BCa (17 cases), G2-grade BCa (16 cases), G3-grade BCa (15 cases), and metastatic lymph nodes (2 cases). We found that the Cofilin1 protein was localized mainly in the epithelial cell membrane and cytoplasm of BCa cells, with low expression in the nucleus and mesenchyme. Positive expression was indicated by a brownish-yellow or brown color. The positive Cofilin1 expression rate was 60.4% (29/48) in the BCa tissues and 0% (0/8) in the normal bladder tissues. The amount of Cofilin1 protein expressed in BCa tissues and normal bladder tissues increased with increasing pathological BCa grade in the lymph nodes ( Figure 1D). Furthermore, in BCa cell lines, Cofilin1 was expressed normally in bladder epithelial (SV-HUC-1) cells, at a slightly higher level in superficial BCa (RT4 and BIU-87) cells, and at a significantly higher level in invasive BCa (EJ and T24) cells, especially T24 cells. We also assessed Cofilin1 protein levels in three cell lines (SV-HUC-1, T24, and RT4) and found that they were significantly higher in the T24 and RT4 cell lines than in the SV-HUC-1 cell line ( Figure 1E,F). Hematoxylin-eosin staining revealed chemotaxis by columnar cells in von Brunn’s nest in cases of adenoid cystitis. In invasive uroepithelial carcinoma, grade 1 tumors presented heterogeneous uroepithelium with nested infiltrative growth and tumor cells with irregular nuclei and visible nuclear divisions, grade 3 tumors appeared markedly heterogeneous, with pleomorphic tumor cells showing irregular nested infiltrative growth and nuclear divisions as well as readily visible tumor necrosis, and grade 2 tumors were intermediate between Grades 1 and 3 ( Figure 1G). These findings indicated that Cofilin1 was highly expressed in BCa tissues and cell lines, and localized mainly in the cytoplasm of BCa cells, and that its expression level increased with increasing pathological grade of BCa.

### Cofilin1 promotes the invasion and metastasis of BCa cells *in vitro* and *in vivo*

**Figure 2:**
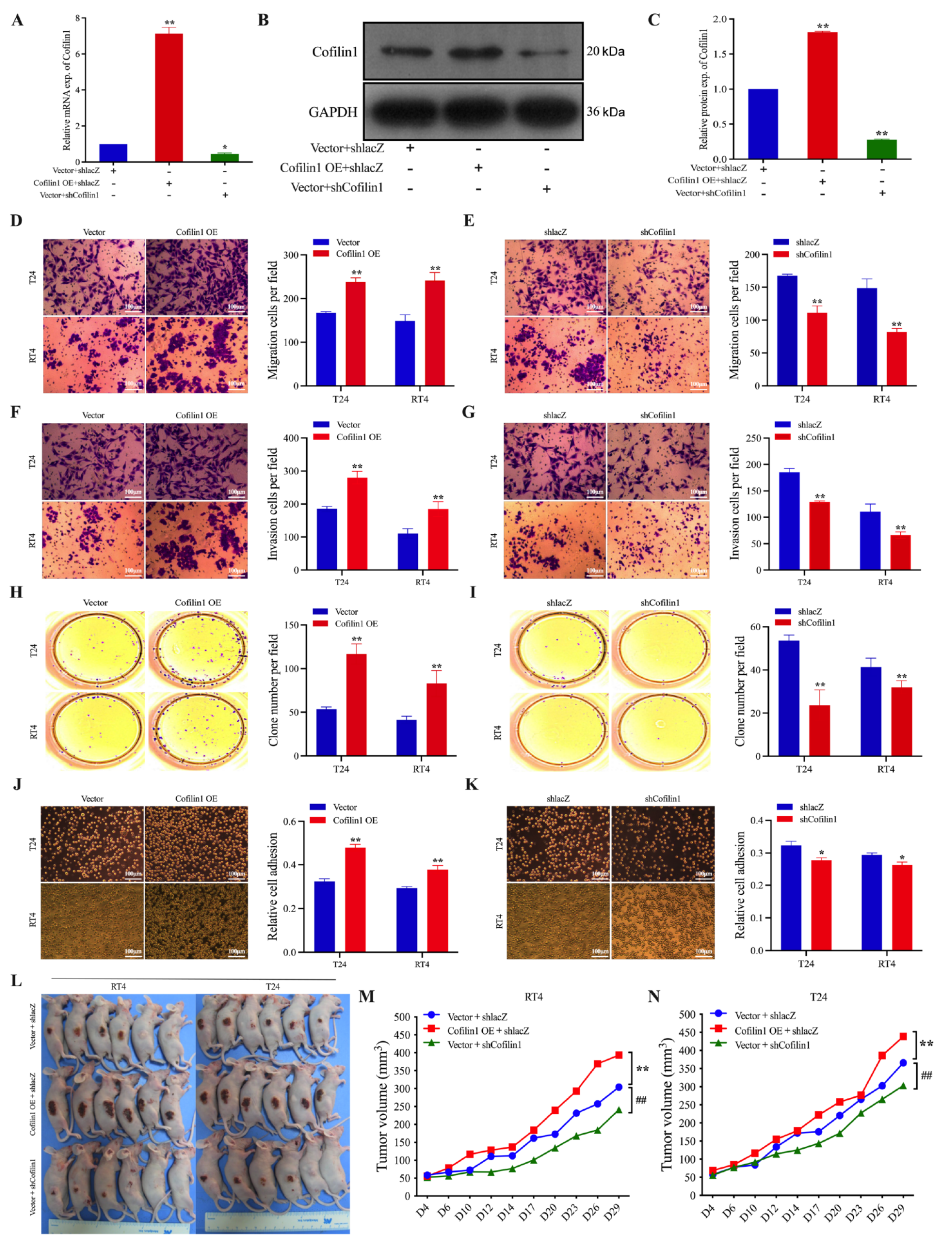
Figure 2 Stable overexpression of Cofilin1 promotes the malignant phenotype of BCa cells *in vitro* and *in vivo* (A–C) qRT-PCR and western blot analysis were performed to detect the transfection efficiency of Cofilin1 OE and shCofilin1 in the constructed T24 cell line, respectively (n = 3). (D–K) The effects of stable overexpression and silencing of Cofilin1 on T24 and RT4 cell migration (D,E), invasion (F,G), colony formation (H,I) and cell adhesion (J,K) were determined. Scale bar = 100 μm. n = 3. (L–N) Final tumor sizes and corresponding statistical plots of subcutaneous xenograft tumors formed from subcutaneous xenograft tumors stably transfected with RT4 cells and T24 cells by Cofilin1 OE or shCofilin1, respectively (n = 6). *P < 0.05, **P < 0.01. Exp., expression.

To investigate the effects of Cofilin1 on the invasion and metastasis of BCa, we transfected BCa cells with a shRNA (shCofilin1) or a plasmid (Cofilin1 OE) to silence or overexpress *Cofilin1*, respectively. We then confirmed the transfection efficiency of the siRNAs and plasmids via qRT-PCR and western blot analysis. The expression of Cofilin1 was significantly greater in the Cofilin1 OE group than in the vector + shlacZ group and significantly lower in the shCofilin1 group ( *P*  < 0.01 and *P*  < 0.05, respectively; Figure 2A–C), indicating that the Cofilin1 overexpression and silencing vectors were successfully constructed *in vitro* and were stably expressed in BCa cells. Next, we examined the effect of Cofilin1 on the proliferation of BCa cells. Transwell and colony formation assays revealed that the numbers of migrating and invading BCa cells were significantly greater in the group that overexpressed Cofilin1 than in the control group, whereas knockdown of *Cofilin1* significantly reduced the number of migrating and invading BCa cells ( Figure 2D–G). Furthermore, we examined the effect of Cofilin1 on the proliferation of BCa cells via a colony formation assay to detect the colony formation ability of T24 and RT4 cells. We found that the overexpression of Cofilin1 promoted the formation of T24 and RT4 cell clones, whereas the silencing of *Cofilin1* significantly inhibited the formation of these clones ( Figure 2H,I). Moreover, a cell adhesion assay was performed to observe the adhesion of T24 and RT4 cells to fibronectin. The results indicated that the overexpression of Cofilin1 increased the ability of BCa cells to adhere to fibronectin and that the silencing of *Cofilin1* decreased this ability ( Figure 2J,K). These findings suggest that Cofilin1 promotes the growth and proliferation of BCa cells.

To further investigate the *in vivo* effects of Cofilin1 on BCa, we established a xenograft tumor model using T24 and RT4 cells in BALB/C-NU nude mice. Before transplantation, T24 and RT4 cells were transfected with the Cofilin1 expression vector, the *Cofilin1* silencing vector, or the control vector, followed by subcutaneous transplantation into female mice. Tumor dimensions were monitored every 3 days until day 29, when the mice were euthanized by cervical dislocation for tumor tissue collection. Compared with control tumors, *Cofilin1*-silenced tumors presented significantly slower growth kinetics and smaller final volumes. Conversely, Cofilin1-overexpressing tumors displayed accelerated growth with markedly increased volumes ( Figure 2L–N). In parallel with the lung metastasis models, Cofilin1 overexpression substantially enhanced pulmonary metastatic nodule formation, whereas *Cofilin1* silencing effectively reduced the metastatic burden compared with that in the control groups ( Supplementary Figure S1). These findings collectively indicate that Cofilin1 promotes both the tumorigenesis and metastasis of BCa *in vitro* and *in vivo*.

### Upregulation of Cofilin1 expression promotes the formation of invadopodia in BCa cells

**Figure 3:**
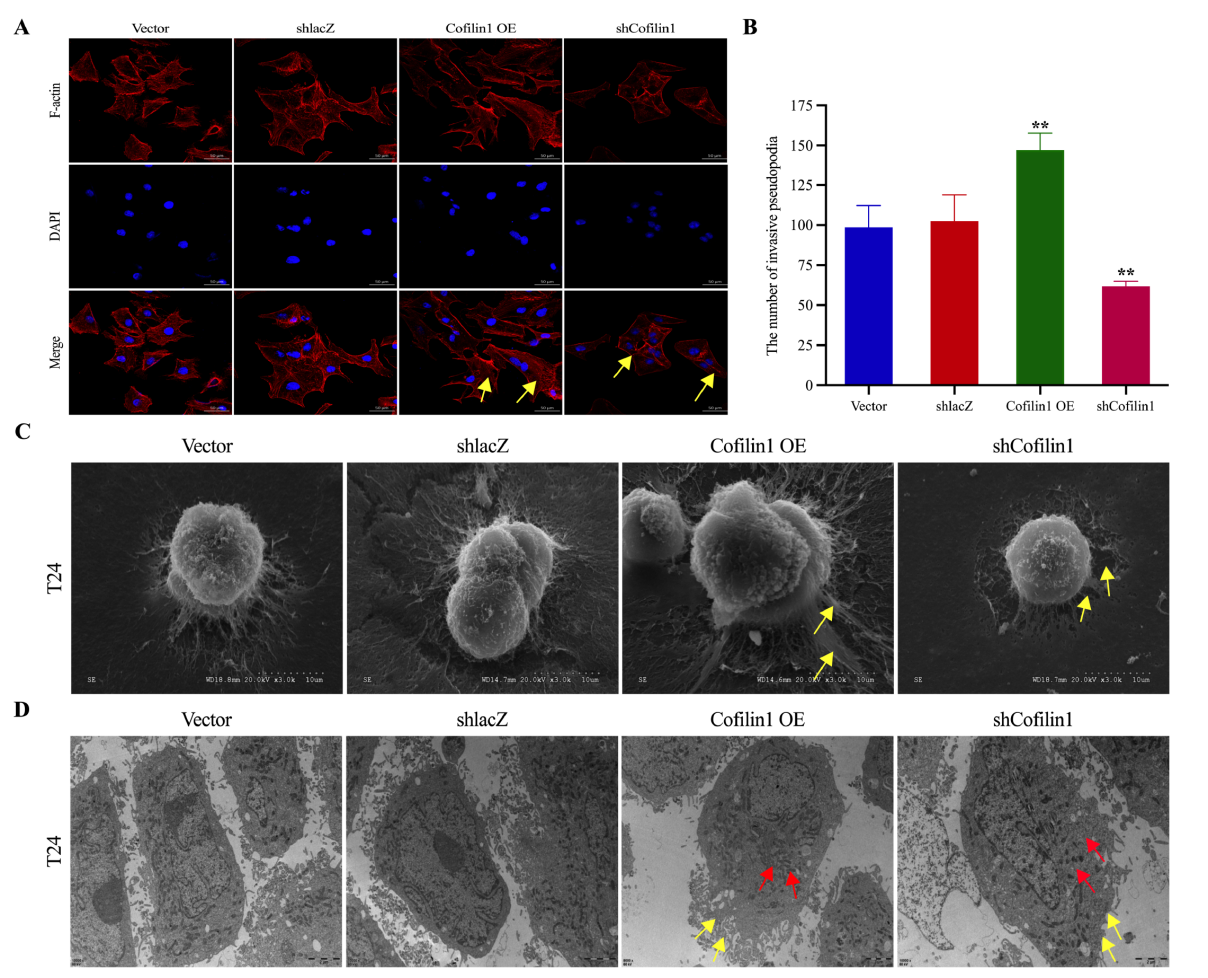
Figure 3 Elevated Cofilin1 expression promotes the formation of invadopodia in BCa cells (A) Immunofluorescence detection of F-actin to observe the formation of invadopodia in bladder cancer T24 cells. Scale bar = 50 μm. n = 3. (B) Statistical graph of the number of invadopodia of BCa cells in different treatment groups. (C) Scanning electron microscopy was used to observe the morphology and fine structure of BCa cell invadopodia in different treatment groups. Scale bar = 10 μm. n = 3. (D) Distribution of bladder cancer T24 organelles was observed by transmission electron microscopy after overexpression and silencing of Cofilin1. Scale bar = 2 μm. n = 3. Yellow arrow: the formation of invadopodia; red arrow: the number of mitochondria. **P < 0.01.

The formation of invadopodia usually degrades the surrounding stroma and promotes distal invasion of cancer cells [15]. After we confirmed that Cofilin1 promoted the migration and invasion of BCa cells, our next step was to clarify the effect of Cofilin1 on the formation of invadopodia in these cells. We established a control group, a Cofilin1 OE group, and an shCofilin1 group, after which we observed the formation of invadopodia and the microstructure of the surface of BCa cells by staining for changes in the cytoskeleton with the invadopodia-related protein F-actin via a ghost pen cyclic peptide and by scanning electron microscopy. Compared with those in the control group, the number of plate-like invadopodia and stress fibres increased in Cofilin1-overexpressing T24 cells, whereas the silencing of *Cofilin1* resulted in a significant decrease in the amount of F-actin ( Figure 3A). Interestingly, scanning electron microscopy revealed that the invadopodia on the ventral side of Cofilin1-overexpressing T24 cells were thicker and more numerous and that the subcellular stromal gel appeared to be markedly disrupted by the adhesion and degradation of invadopodia, with extensive and tight multicellular contacts. In contrast, after *Cofilin1* was silenced, T24 cells presented atypical growth of surface microvilli, small and few invadopodia on the ventral side of the cells, and slight degradation and disruption of the subcellular matrix ( Figure 3C).

It is well known that cells in aggressive tumors usually need an increased energy supply for growth and metastasis. Metabolic reprogramming ( *e*. *g*., β-oxidation of fatty acids in mitochondria) is rapidly initiated as a compensatory pathway for the proliferation, invasion, and metastasis of hypermetabolic cancer cells [16]. Therefore, we observed the distribution of organelles by high-resolution transmission electron microscopy. We found that T24 cells in the control group were surrounded by more microvilli and filamentous invadopodia and had more mitochondria around the nucleus and an irregular nucleolus morphology. However, after overexpression of Cofilin1, there was a significant increase in the number of microvilli and filamentous invadopodia around the cells as well as a significant increase in the number of mitochondria around the nucleus and irregular nucleolus morphology. In contrast, after *Cofilin1* was silenced, there were fewer microvilli and filamentous invadopodia on the surface of the T24 cells, which had irregularly shaped nuclei and significantly fewer mitochondria ( Figure 3D). Statistical analysis revealed that the number of invadopodia in BCa cells significantly increased after Cofilin1 was overexpressed and significantly decreased after *Cofilin1* was knocked down ( Figure 3B). These findings indicate that high expression of Cofilin1 promotes the formation of invadopodia in BCa cells and leads to a significant increase in the number of mitochondria and increased metabolism in these cells.

### Cofilin1/Cortactin synergistically promotes the formation of invadopodia in BCa cells

**Figure 4:**
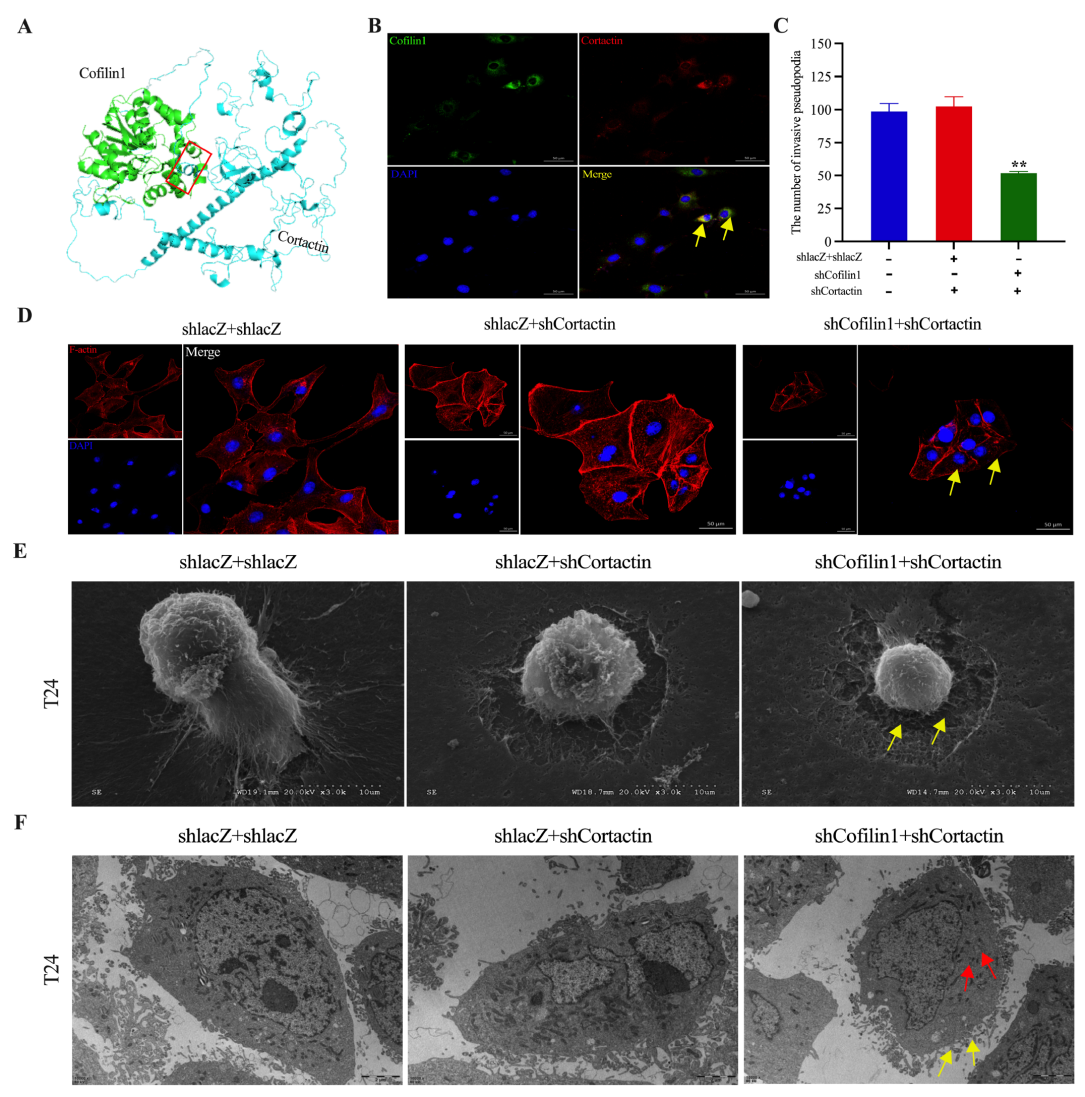
Figure 4 Cofilin1/Cortactin synergy promotes invadopodia formation in BCa (A) Bioinformatics analysis predicted a possible structural domain of protein interaction between Cofilin1 and Cortactin. Green represents Cofilin1 and blue represents Cortactin. (B) Immunofluorescence staining verified the interaction and co-localisation of Cofilin1 and Cortactin in bladder cancer T24 cells. Scale bar = 50 μm. n = 3. (C) Statistical graph of the number of invadopodia of BCa cells in different treatment groups. (D) Immunofluorescence detection of shCofilin1 and shCortactin simultaneously transfected with F-actin in bladder cancer T24 cells to observe the formation of invadopodia. Scale bar = 50 μm. n = 3. (E) Scanning electron microscopy was used to observe the morphology and fine structure of invadopodia in shCofilin1 and shCortactin simultaneously transfected T24 cells. Scale bar = 10 μm. n = 3. (F) Distribution of organelles in T24 cells was observed by transmission electron microscopy after silencing of Cofilin1 and Cortactin. Scale bar = 2 μm. n = 3. Yellow arrow: the formation of invadopodia; red arrow: the number of mitochondria. **P < 0.01.

Cortactin is a protein closely related to the cytoskeleton and cell motility and plays an important role in regulating the formation of invadopodia and morphological changes in cancer cells by binding to actin and microfilaments [17]. However, whether Cofilin1 promotes the formation of invadopodia in BCa cells by binding to Cortactin proteins is not clear. Therefore, we identified the likely structural helix-helix interaction domain between Cofilin1 and Cortactin using the online tool ClusPro 2.0 for analysis of the protein‒protein docking structure ( Figure 4A). Immunofluorescence detection was then used to confirm the colocalization of Cofilin1 and Cortactin in the cytoplasm of BCa cells, which suggested mutual binding of the two proteins to synergize their protein functions ( Figure 4B). After downregulating the expressions of Cofilin1 and Cortactin by simultaneous transfection of BCa T24 cells with shCofilin1 and shCortactin, we detected the expression of F-actin by immunofluorescence staining and observed the formation of invadopodia in BCa cells by scanning electron microscopy. We found that the amount of F-actin was significantly lower in BCa cells in which both *Cofilin1* and *Cortactin* were silenced than in the blank control group ( Figure 4C,D).

Notably, scanning electron microscopy revealed atypical growth of microvilli on the surface of T24 cells after simultaneous silencing of *Cofilin1* and *Cortactin* and that the invadopodia on the ventral side of the cells were thinned and significantly fewer in number, with significant degradation and disruption of the subcellular matrix ( Figure 4E). Moreover, high-resolution transmission electron micrographs revealed that the numbers of microvilli on the cell surface and filamentous invadopodia were reduced further after knockdown of both *Cofilin1* and *Cortactin*, the nuclei were irregularly shaped, and the number of mitochondria was lower ( Figure 4F). These findings indicated that Cofilin1 and Cortactin synergistically remodeled the F-actin cytoskeleton and promoted the formation of invadopodia in BCa cells.

### Expression levels and clinical correlation analysis of TCF7L2, miR-206 and Cofilin1 in BCa invasive metastasis

Further exploration of the *Cofilin1* gene as a target for bladder cancer treatment provides a new basis. First, we used the TCGA and cbioPortal gene databases to detect the correlation between the expression levels and clinical prognosis of TCF7L2, miR-206 and Cofilin1 in clinical BCa patients. We collected correlation data for TCF7L2 and Cofilin1 but did not find correlation data for miR-206, so we analyzed the expressions of TCF7L2 and Cofilin1 in BCa invasion and metastasis in relation to clinical prognosis. The results revealed that Cofilin1 expression was significantly increased in BCa tissues compared with normal bladder tissues, whereas TCF7L2 expression was decreased in BCa tissues ( Supplementary Figure S2A,B). There was no correlation between *Cofilin1* and *TCF7L2* gene expressions in BCa tissues ( Supplementary Figure S2C). Moreover, we analyzed the expression levels of *the Cofilin1* and *TCF7L2* genes across cancers by mining the TCGA and GEO databases via the online analysis tool TIMER and found that the expression of *Cofilin1* mRNA was greater in BCa tissues than in bladder tissues in the TCGA and GEO series ( Supplementary Figure S2D), whereas the *TCF7L2* mRNA expression levels were significantly lower ( Supplementary Figure S2F). Interestingly, however, the use of the GEPIA online tool to analyze survival prognosis revealed that the expression levels of Cofilin1 and TCF7L2 were associated with OS and DFS in BCar patients, with higher expression of Cofilin1 and TCF7L2 resulting in shorter OS and DFS in patients, and vice versa in the low-expression group ( Supplementary Figure S2E,G). In addition, we constructed a risk score model for BCa samples on the basis of the expression and regression coefficients of the *Cofilin1* and *TCF7L2* genes and divided the samples into a high-risk score group ( *n* = 86) and a low-risk score group ( *n* = 85). The results of the visualization analysis revealed that red represents the high-risk score group, and blue represents the low-risk score group ( Supplementary Figure S2H,K). As shown in the figure, patients in the high-risk group had higher morbidity and mortality rates than those in the low-risk group did, suggesting that the high-risk group was associated with a poor prognosis ( Supplementary Figure S2I,L). While Cofilin1 and TCF7L2 were highly expressed in the high-risk scoring group, the high expression of these 2 genes, *Cofilin1* and *TCF7L2*, was positively correlated with high risk, as shown by the time-dependent curve ROC results ( Supplementary Figure S2J): the AUC at 1 year was 0.615, the AUC at 3 years was 0.609, and the AUC at 5 years was 0.539.

To evaluate the prognostic significance of the clinical and molecular variables, multivariate Cox proportional hazards regression models were applied. The results revealed that Cofilin1 expression level [log₂(HR) = 0.708, 95% CI: 0.028–1.38; *P* = 0.041] and neoadjuvant therapy [log₂(HR) = 2.98, 95% CI: 1.06–4.75; *P* = 0.002] were independent predictors of patient survival. Cofilin1 was suggested as a potential risk factor, while neoadjuvant therapy may significantly increase the risk ( Supplementary Figure S3A,B). In contrast, TCF7L2 expression was not significantly associated with survival outcomes in any model ( Supplementary Figure S3C,D). These findings indicate that the BCa risk scoring model has robust predictive performance for prognosis. Furthermore, the data suggest that Cofilin1 may serve as a potential biomarker for predicting metastatic outcomes in BCa patients.

### *TCF7L2* knockdown inhibits the proliferation and metastasis of BCa cells

**Figure 5:**
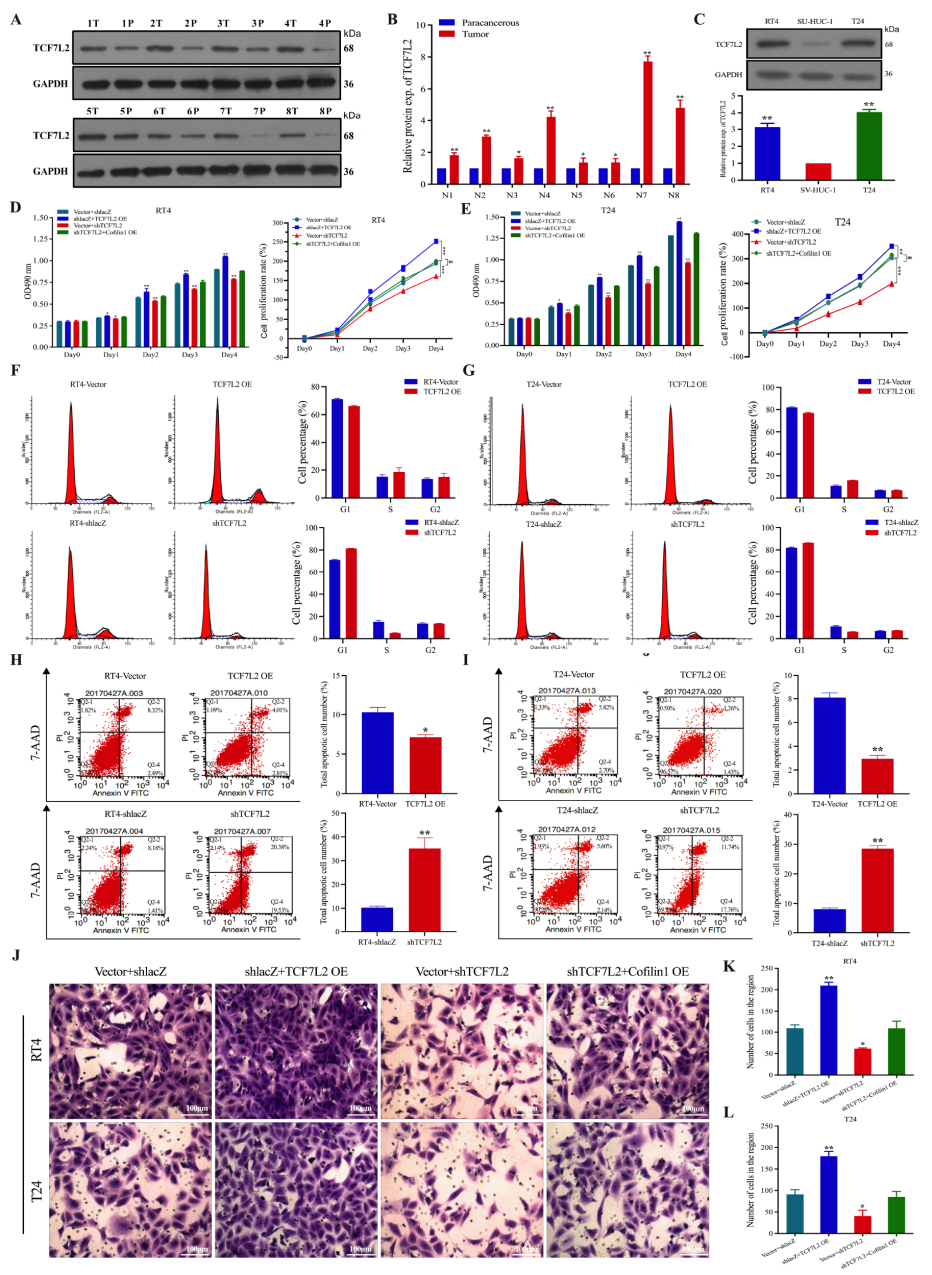
Figure 5 *TCF7L2* knockdown inhibits BCa cell proliferation, migration and cell cycle, and promotes apoptosis (A,B) Protein expression and statistical graphs of TCF7L2 in BCa tissues and paracancerous tissues of 8 patients detected by western blot analysis, and GAPDH was used as a loading control (n = 8). (C) Western blot analysis was used to determine the protein expression level of TCF7L2 in SV-HUC-1, RT4 and T24 cell lines (n = 3). (D,E) The effects of stable overexpression and inhibition of TCF7L2 on the proliferation of RT4 and T24 cells (n = 3). (F,I) After overexpression or inhibition of TCF7L2, cell cycle changes and apoptosis were detected by flow cytometry in RT4 and T24 cells, respectively (n = 3). (J–L) Transwell assays of RT4 and T24 cell migration after overexpression or knockdown of TCF7L2. Scale bar = 100 μm. n = 3. *P < 0.05, **P < 0.01. Exp., expression.

Western blot analysis of eight patients with BCa revealed that TCF7L2 expression was significantly upregulated in cancerous tissues compared with paracancerous tissues. The western blot analysis results also confirmed that the protein expression of TCF7L2 was elevated in BCa cells ( Figure 5A–C). To further elucidate the role of TCF7L2 in BCa, vectors and plasmids containing siRNA sequences specifically targeting the reverse splicing region of TCF7L2 were stably transfected into RT4 and T24 cells to inhibit or upregulate the expression of TCF7L2. We examined the effects of disruption of *TCF7L2* on the proliferation of BCa cells. The results of the CCK-8 assay revealed that overexpression of TCF7L2 accelerated the proliferation of RT4 and T24 cells, whereas knockdown of *TCF7L2* significantly inhibited the growth of these cells, and these effects were reversed when Cofilin1 was simultaneously overexpressed ( Figure 5D,E). However, the regulatory relationship between TCF7L2 and Cofilin1 in the metastasis of BCa remains to be explored in depth. Next, we examined the effect of TCF7L2 on the cell cycle in BCa cells by flow cytometry. The results revealed that knockdown of *TCF7L2* increased the percentage of cells in the G2 phase, indicating cell cycle arrest, and significantly decreased the proportion of cells in an active proliferative state ( Figure 5F,G). We then investigated whether changes in apoptosis could explain this phenomenon and found that the total apoptosis rate of RT4 and T24 cells was significantly increased after the knockdown of *TCF7L2* and significantly decreased after the overexpression of TCF7L2 ( Figure 5H,I). Furthermore, a transwell migration assay revealed that stable overexpression of TCF7L2 promoted significant migration and invasion of BCa cells ( Figure 5J–L). These results suggest that *TCF7L2* deficiency attenuates the growth and proliferation of BCa cells, leading to cell cycle arrest, apoptosis, and the inhibition of BCa progression.

### TCF7L2 promotes the expression of Cofilin1 by inhibiting the transcription of miR-206

**Figure 6:**
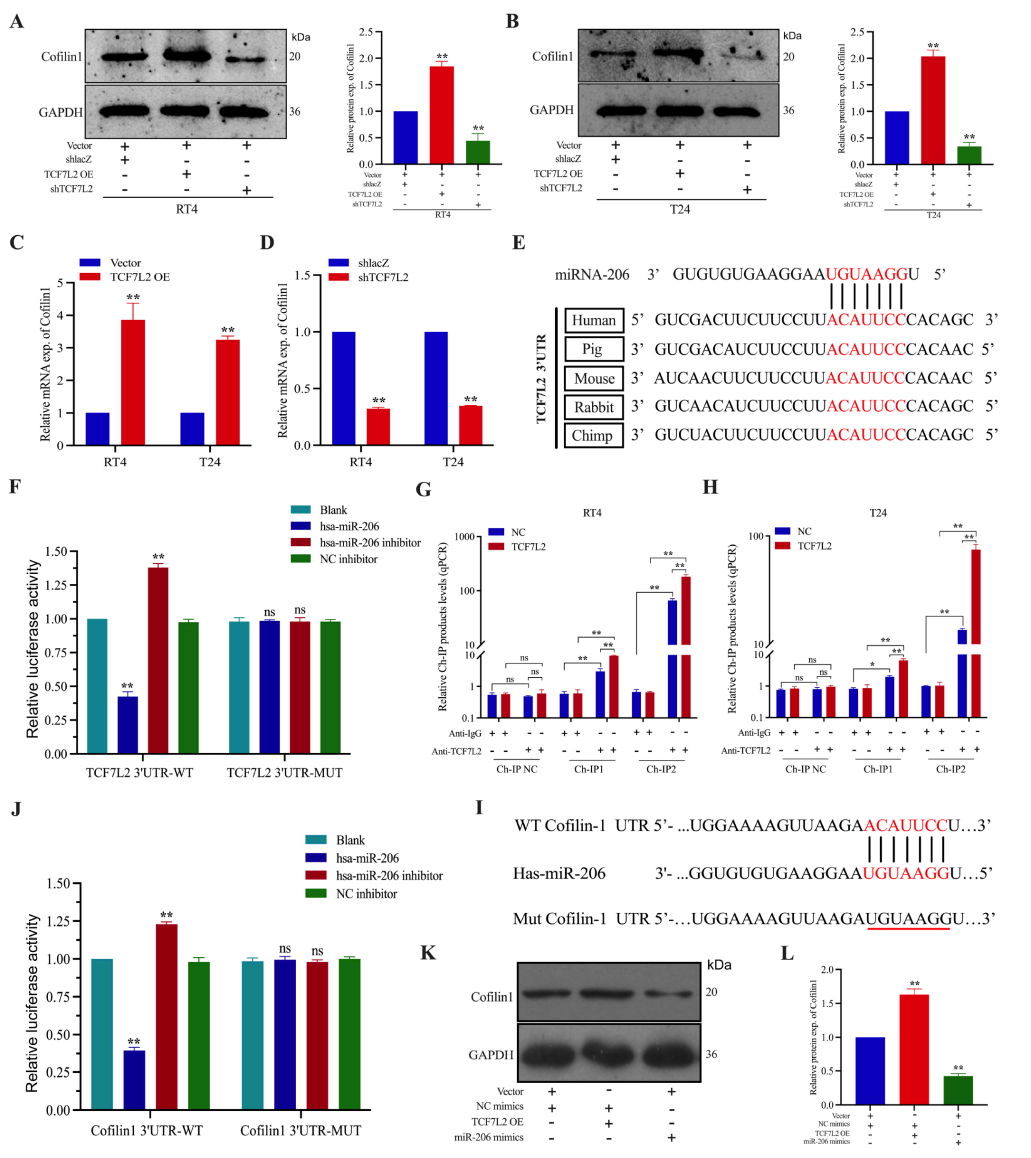
Figure 6 TCF7L2 promotes Cofilin1 expression by inhibiting miR-206 transcription (A,B) Western blot analysis of Cofilin1 protein expression in BCa cells with TCF7L2 overexpression/knockdown (n = 3). (C,D) qRT-PCR was used to detect the effect of TCF7L2 overexpression/knockdown on Cofilin1 transcription in BCa cells (n = 3). (E) Schematic representation of miR-206 binding site of TCF7L2 in different species. (F) Dual luciferase assay in 293 T cells cotransfected with TCF7L2-WT (or TCF7L2-Mut) promoter plasmid and miR-206 overexpression plasmid (or empty vector) (n = 3). (G,H) Ch-IP assay verifies direct binding of RT4 to the miR-206 promoter and TCF7L2 in T24 cells. Data are expressed as the mean ± SD of three independent experiments. *P < 0.05; **P < 0.01. N.S., not significant. (I) Predicted binding site of miR-206 to Cofilin1 is shown. (J) Dual luciferase assay for targeted binding between both miR-206 and Cofilin1 (n = 3). (K,L) Western blot analysis was used to detect Cofilin1 expression after overexpression of TCF7L2 or miR-206 in BCa cells (n = 3). **P < 0.01. Exp., expression.

Next, to investigate the regulatory relationship between TCF7L2 and Cofilin1, *TCF7L2* was knocked down or overexpressed in BCa cell lines to determine its effect on the transcription and expression of *Cofilin1*. Western blot analysis and qRT-PCR revealed that overexpression of TCF7L2 significantly increased the transcription and expression of Cofilin1 in T24 and RT4 cells, whereas shTCF7L2 had the opposite effect ( Figure 6A‒D). However, the mechanism by which TCF7L2, as a transcription factor, regulates the expression of Cofilin1 to promote the metastasis of BCa needs further investigation. Bioinformatics analysis revealed that TCF7L2 has potential binding sites for Hsa-miR-206 ( Figure 6E). We used a dual-luciferase reporter gene system to clarify whether TCF7L2 binds to miR-206 and found that the luciferase activity of the TCF7L2-WT promoter could be significantly activated by co-transfection of miR-206 in HEK-293T cells; importantly, transfection of the Hsa-miR-206 inhibitor resulted in diminished luciferase activity at the TCF7L2-WT promoter. However, activation was significantly attenuated when the TCF7L2 binding site for miR-206 was mutated ( Figure 6F). ChIP analysis further confirmed the specific binding of TCF7L2 to the miR-206 promoter region, with two genomic intervals (–1777 to –1532 bp and –743 to –570 bp) showing significant enrichment compared with the IgG controls ( Figure 6G,H).

Further analysis of the miR-206 target genes using bioinformatics tools such as TargetScan revealed a TCF7L2-binding site in the promoter region of the miR-206 gene, which was consistent with our previous findings and suggested that *Cofilin1* is a potential target gene downstream of miR-206 ( Figure 6I). This finding was confirmed by the luciferase reporter gene system; that is, luciferase activity was elevated in T24 cells cotransfected with a luciferase reporter gene containing the wild-type *Cofilin1* mRNA 3′UTR fragment and the miR-206 inhibitor, whereas co-transfection of Cofilin1 containing a mutation in the miR-206-binding site mRNA 3′UTR fragment of the luciferase reporter gene and the miR-206 inhibitor did not lead to any change in luciferase activity in cells ( Figure 6J). Western blot analysis revealed that overexpression of miR-206 decreased Cofilin1 protein level, and conversely, overexpression of TCF7L2 upregulated Cofilin1 protein level ( Figure 6K,L), suggesting that miR-206 targets and inhibits the expression of Cofilin1. These experimental data suggest that TCF7L2 may reverse the inhibitory effect of miR-206 on Cofilin1 expression by inhibiting miR-206 transcription. Furthermore, to further evaluate the anti-proliferative and anti-invasive effects of miR-206, we established stable cell lines with miR-206 overexpression or knockdown. Functional assays demonstrated that miR-206 overexpression significantly suppressed cell proliferation ( *P*  < 0.01), migration ( *P*  < 0.001), and invasion ( *P*  < 0.001). Conversely, miR-206 knockdown markedly enhanced these oncogenic processes ( Supplementary Figure S4).

### TCF7L2 and miR-206 form an RNA-induced silencing complex

**Figure 7:**
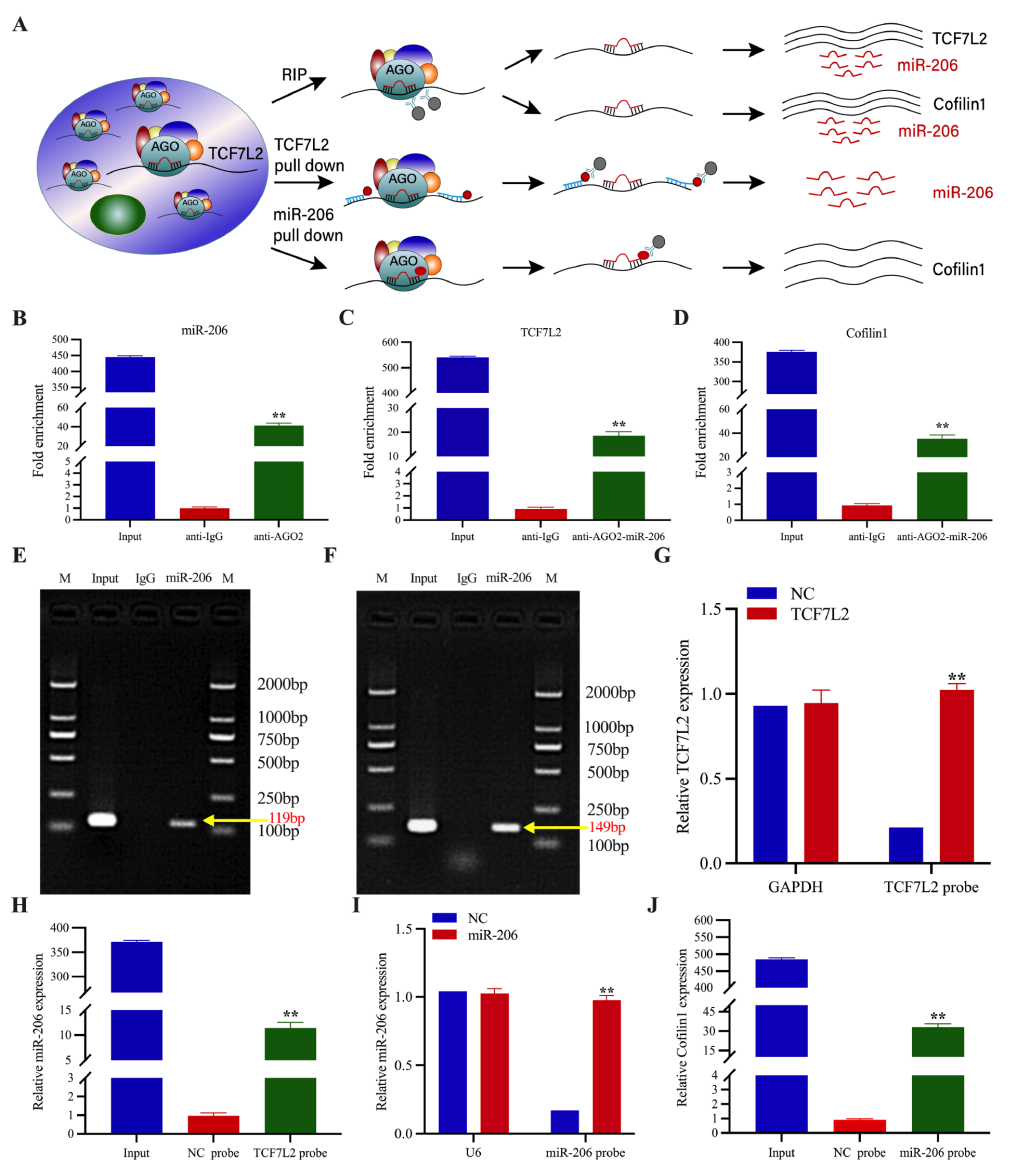
Figure 7 TCF7L2 and miR-206 form an RNA-induced silencing complex (A) Scheme of the pull-down assay used to validate the physical interaction between TCF7L2/miR-206/Cofilin1. (B–D) RNA immunoprecipitation in T24 cells using anti-AGO2 antibody. Anti-IgG antibody was used as a control (n = 3). (E,F) Agarose gel electrophoresis was performed to detect the TCF7L2/Cofilin1 PCR products of the samples to be tested, respectively. (G,I) RNA pull-down was performed in T24 cells using 50 biotin-labelled TCF7L2 RNA probe and hsa-miR-206 probe, respectively. Enrichment of TCF7L2 and miR-206 was detected by qRT-PCR (n = 3). (H) RNA pull-down using 50 biotin-labelled TCF7L2 probes in T24 cells and detection of enrichment of this probe for miR-206 (n = 3). (J) RNA pull-down using 50 biotin-labelled miR-206 probes in T24 cells and detection of enrichment of this probe for Cofilin1. Data are presented as the mean ± SD, n = 3. **P < 0.01.

To further elucidate the mechanism of the transcriptional regulation of miR-206 by TCF7L2, we observed the binding of TCF7L2/miR206 and miR206/Cofilin1 via RNA immunoprecipitation experiments (using an AGO2 antibody). We used RNA pull-down to capture RNA with a TCF7L2 probe for detection of miR-206 and detected Cofilin1 using the miR-206 probe to capture RNA for Cofilin1. The Argonaute (Ago) family of proteins was first identified in *Arabidopsis thaliana* by Bohmert *et al*. [18], who reported that these proteins play important roles in the biogenesis and function of miRNAs. miRs target RNAs, and AGO2 forms an RNA-induced silencing complex (RISC) to mediate miR-induced gene silencing [19]. Given that endogenous expression of miR-206 is relatively low in BCa tissues and cells, we investigated whether TCF7L2 and miR-206 are present in the AGO2-miR-206-TCF7L2 complex via the use of miR-206-overexpressing T24 for RNA immunoprecipitation and TCF7L2/miR-206 and miR206/Cofilin1 pull-down assays ( Figure 7A). First, we performed RNA immunoprecipitation using a specific anti-AGO2 antibody to pull down RNA that interacts with AGO2 proteins. Mature miR-206 was successfully enriched ( Figure 7B), indicating that miR-206 interacts with AGO2. RNA immunoprecipitation with anti-AGO2-miR-206 was then performed to pull down the proteins that interact with AGO2; both TCF7L2 and Cofilin1 were found to be significantly enriched ( Figure 7C–F), indicating that TCF7L2 and Cofilin1 interact with miR-206. Next, we investigated whether there is a mutual physical binding effect between TCF7L2, miR-206 and Cofilin1. Fifty biotin-labelled RNA probes were used to pull down miR-206 and Cofilin1. TCF7L2 transcripts were enriched by the TCF7L2-specific probe ( Figure 7G), suggesting that this probe could successfully pull down TCF7L2. Compared with the control probe, specific binding via the TCF7L2 probe was significantly enriched in mature miR-206 ( Figure 7H). Similarly, 50 biotin-labelled Hsa-miR-206 probes were pulled down and enriched with Cofilin1 compared with the control probes ( Figure 7I–J). These data suggest that TCF7L2 directly interacts with miR-206 and AGO2 to form a RISC.

### TCF7L2/miR-206 regulate Cofilin1 to promote invasion and metastasis of BCa

**Figure 8:**
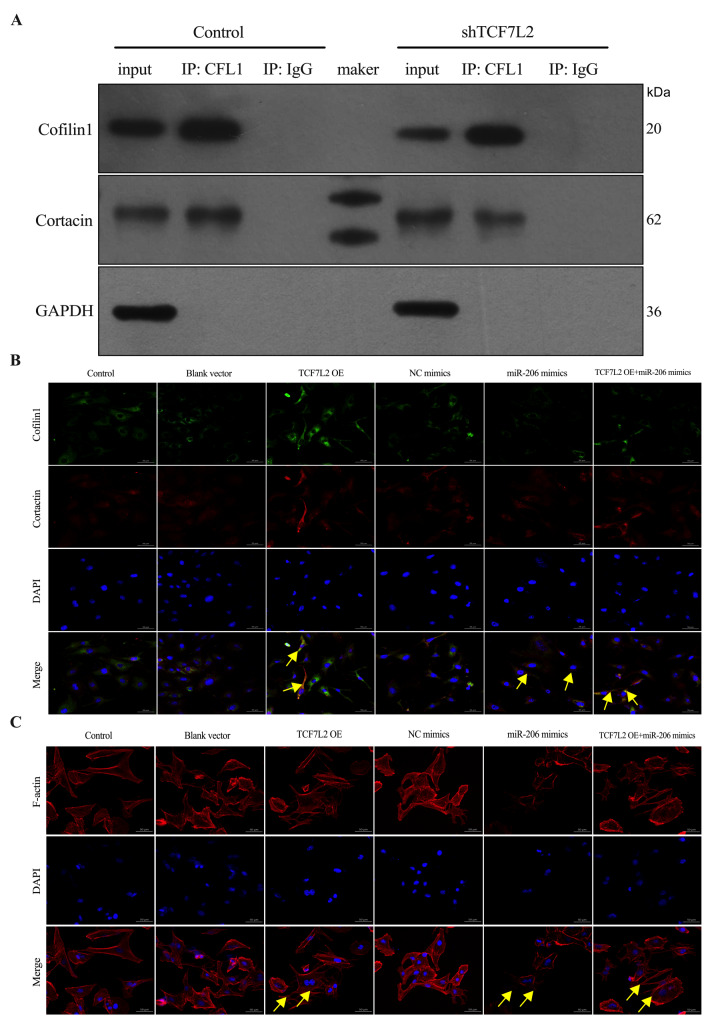
Figure 8 TCF7L2 inhibits miR-206 transcriptional maturation regulating Cofilin1 to promote invasive metastasis of BCa (A) Co-IP was performed to determine the effect of TCF7L2 on the binding between Cofilin1 and Cortactin in T24 cells (n = 3). (B) Immunofluorescence staining was performed to observe the changes of Cofilin1 co-localisation with Cortactin in T24 cells by overexpression of TCF7L2 and miR-206. Scale bar = 50 μm. n = 3. (C) Immunofluorescence staining was used to detect the effect of overexpression of TCF7L2 and miR-206 on the formation of invadopodia in bladder cancer in T24 cells. Scale bar = 50 μm. Data are presented as the mean ± SD, n = 3. **P < 0.01.

Previously, we confirmed co-localization of Cofilin1 and Cortactin in the cytoplasm of T24 BCa cells. Next, we investigated the interaction between Cofilin1 and Cortactin by exogenous co-immunoprecipitation. The results revealed that Cofilin1 interacted with Cortactin in HEK-293T cells and that the binding between Cofilin1 and Cortactin was significantly attenuated after *TCF7L2* was knocked down compared with that in the control group ( Figure 8A). An immunofluorescence staining assay in T24 cells confirmed this complementary pattern. We found that co-localization of both Cofilin1 and Cortactin was promoted by overexpression of TCF7L2 and significantly reduced by overexpression of miR-206, whereas simultaneous overexpression of TCF7L2 significantly attenuated the binding of Cofilin1 to Cortactin. The inhibitory effect of miR-206 on co-localization of Cofilin1 and Cortactin was also observed ( Figure 8B). These findings indicate that TCF7L2 regulates the co-localization of Cofilin1 with Cortactin by inhibiting the maturation of miR-206.

Cofilin1 is an actin-binding protein that promotes cell migration and motility via the polymerization/depolymerization of actin fibers, which changes the degree of adhesion between cells and the extracellular matrix [20]. However, cell migration is driven by targeted polymerization of F-actin, which leads to local protrusions in the cell membrane [21]. Therefore, we used a ghost pen cyclic peptide to stain F-actin and observed cytoskeletal changes in cancer cells via laser confocal microscopy to further clarify the roles of TCF7L2 and miR-206 in the formation of invadopodia in BCa cells. The immunofluorescence staining results revealed that, compared with the empty vector, the overexpression of TCF7L2 increased the amount of F-actin, plate-like invadopodia, and stress fibers. In contrast, overexpression of miR-206 decreased the amount of F-actin, and simultaneous overexpression of TCF7L2 reversed the inhibitory effect of miR-206 on the formation of F-actin ( Figure 8C). These findings suggest that TCF7L2 may promote the invasion and metastasis of BCa by inhibiting the transcriptional maturation of miR-206, thereby increasing the expression of Cofilin1 and promoting the formation of invadopodia in BCa.

## Discussion

BCa is a highly invasive malignancy that is life-threatening and has a serious impact on quality of life [22]. In-depth research on the mechanism by which BCa develops and metastasizes is important for its prevention and treatment. Cofilin1 is a marker of tumor progression and is considered a “metastatic switch.” Prevalent in eukaryotic organisms, it belongs to the actin depolymerization factor/Cofilin superfamily, the members of which are important regulators of depolymerization and polymerization of actin and reorganization of the cytoskeleton and are known to be upregulated in radiotherapy-resistant tumors [ 23, 24] . Furthermore, reorganization of the actin cytoskeleton is necessary for the invasion of neighboring tissues by tumor cells, with the formation of invadopodia and generation of forces that drive cell migration and infiltration. Upregulation of Cofilin1 induces remodeling of the F-actin cytoskeleton, accelerates the proliferation and migration of cells, and promotes the formation of clones, thereby promoting resistance. Knockdown of the *Cofilin1* gene can block signal transduction and inhibit cell migration and may be an effective therapeutic strategy for radiotherapy-resistant tumors. Some studies have shown a close association between the activation of Cofilin1 and the invasiveness of tumor cells; the inhibition of Cofilin1 activity reduces the motility and migration of tumor cells and may serve as an important target for inhibiting the growth and proliferation of tumors [25]. In recent years, there has been a strong focus on the role of the expression and regulation of Cofilin1 in malignant tumors. Cofilin1 is also known to play important regulatory roles in tumorigenesis, development, prognosis, metastasis, and epithelial-mesenchymal transition (EMT) in malignancy and drug resistance. Luo *et al*. [26] explored the role of the PUS10/miR-194-5p/NUDC/Cofilin1 regulatory axis in renal cell carcinoma and reported that HIF1A inhibited PUS10 by promoting the maturation of miR-194-5p to regulate the migration of NUDC/Cofilin1-dependent renal cell carcinoma cells. Guo *et al*. [27] also reported that the long-coding RNA DANCR sponges miR-27a-3p to promote the progression of hepatocellular carcinoma and regulate EMT via the ROCK1/LIMK1/Cofilin1 pathway. Although previous studies have shown that Cofilin1 has both oncogenic and inhibitory effects in many malignancies, little is known about its function in BCa, and how and by what mechanism Cofilin1 regulates the invasiveness of BCa remains unknown. In this study, we systematically investigated the biological function of Cofilin1 in BCa and identified a transcriptional regulatory pattern between TCF7L2/miR-206/Cofilin1. These findings provide a new perspective on the pathogenesis of BCa.

This study revealed that the expression level of Cofilin1 in patients with BCa was significantly greater in cancerous tissues than in normal tissues. Further studies revealed that high Cofilin1 expression was localized mainly in the cytoplasm of BCa cells and that its expression level increased as the pathological stage of BCa progressed. This correlation has also been reported in human glioma, breast, and prostate cancers [ 28– 30] . Tumorigenesis is a multistep process involving the aberrant expression or inactivation of oncogenes. We identified *Cofilin1* as an oncogene in BCa. Deletion of *Cofilin1* significantly inhibited the invasiveness of BCa cells *in vitro* and *in vivo*, whereas overexpression of Cofilin1 resulted in the opposite phenotype. Cell migration is one of the key steps in the invasion and metastasis of malignant tumors. In recent years, many studies have shown that actin-rich structures, such as lamellar pseudopods, filamentous pseudopods, and adhesion spots with polarity, are formed at the anterior end of the cell during motility, after which the protrusions are constantly and repeatedly extended forward to pull the posterior cytosolic circulation forward under the action of contractile forces in the cell [31]. Activation of Cofilin1 is necessary for the migration of tumor cells. Using immunofluorescence co-localization analyses in RT4 and T24 cells and scanning electron microscopy, we investigated the potential mechanisms by which Cofilin1 regulates the proliferation and migration of BCa cells. We found that Cofilin1 was involved in the recruitment of actin fibers to the periphery of the cell through mutual binding with cortactin and that Cofilin1 synergistically promoted the formation of invadopodia in BCa cells, thereby regulating their invasion and migration. Notably, this study revealed the effect of *Cofilin1* knockdown or overexpression on the clonogenic ability of tumor cells, suggesting that this molecule may be involved in the regulation of proliferation to some extent. However, in conjunction with literature reports and key data from this study, such as Transwell invasion assays and invadopodia staining, we propose that the main function of Cofilin1 in BCa is to promote the formation of invadopodia through cytoskeletal remodeling, thereby driving the metastatic process. Changes in clonogenic ability may stem from enhanced cell motility, which increases colony spreading efficiency, or from indirect effects of cytoskeletal dynamics on the cleavage plane. Future studies should further elucidate the independence of the role of Cofilin1 in promoting metastasis and proliferation by specifically targeting its cytoskeletal regulatory function.

Invasion of BCa involves multiple genes and alterations in multiple signaling pathways, and transcription factors in the genome have become a focus of anticancer research because of their roles in oncogenic and oncostatic pathways. Recently, a series of studies on transcription factors and malignant diseases revealed that the transcription factor/miRNA/target gene regulatory axis can influence the formation and progression of tumors via interregulation [32]. TCF7L2 is an important transcription factor in the Wnt signaling pathway and is closely associated with the proliferation and metastasis of various tumors [33]. A previous study by our research group revealed that TCF7L2 regulates the expression of Cofilin1, which in turn promotes the migration and invasion ability of RT4 and T24 cells [34]. To further explore whether the *Cofilin1* gene could be a target for BCa therapy, we systematically analyzed the correlation between the expression levels of TCF7L2 and Cofilin1 in BCa and the clinical prognosis via the online Cancer Genome Atlas Program and cBioPortal databases. Cofilin1 expression was found to be significantly elevated in 406 BCa samples, whereas TCF7L2 expression was decreased. There was no correlation between the expressions of the *Cofilin1* and *TCF7L2* genes in BCa tissues. Interestingly, Cofilin1 and TCF7L2 were significantly associated with clinicopathological parameters and cancer-specific survival in these patients. However, our results suggested that TCF7L2 was elevated in BCa tissues and that its knockdown significantly inhibited the proliferation and migration of BCa cells *in vitro*, while the overexpression of TCF7L2 contributed to the opposite phenotype, which is inconsistent with the conclusions of the online Cancer Genome Atlas (TCGA) and cBioPortal database analyses; thus, we wanted to further explore this phenomenon.

Further bioinformatics analysis revealed that both TCF7L2 and miR-206 are important regulators upstream of Cofilin1, that there is a TCF7L2-binding site in the promoter region of the miR-206 gene, and that the 3′UTR of Cofilin1 contains the binding sequence of miR-206. Therefore, we hypothesized that TCF7L2 may regulate Cofilin1 by binding to miR-206 and inhibiting its transcriptional maturation, leading to a malignant phenotype. Dual-luciferase reporter and ChIP assays revealed that TCF7L2 can bind directly to the promoter of miR-206, which can target and bind to Cofilin1 and inhibit its transcription and expression. The functional regulation of Cofilin1 by TCF7L2 was further confirmed at the protein level. MiR-206 is a miRNA with an oncogenic role in many tumors and is involved in the regulation of the growth, invasion, and metastasis of a wide variety of tumor cells [ 35, 36] . However, whether miR-206 participates as a mediator between TCF7L2 and Cofilin1 in the regulation of invasion and metastasis of BCa still needs further investigation. It has been reported that Argonaute 2 (AGO2) and miR or Mir-target RNA form RISC complexes [ 37,38]. To study whether TCF7L2, miR-206 and AGO2 can form RISC complexes, RIP-PCR and RNA-pulldown experiments were performed to verify the formation of RISC complexes. To investigate whether TCF7L2 and miR-206 are present in the AGO2-miR206-TCF7L2 complex, miR-206-overexpressing T24 cells were subjected to RNA immunoprecipitation and TCF7L2/miR-206 pull-down assays. The results are consistent with previous reports and provide evidence suggesting that Cofilin1 may represent a novel hierarchical species-specific regulator of the occurrence of BCa. We found that TCF7L2 influences the expression of Cofilin1 by counteracting endogenous miR-206, leading to the migration of T24 cells. TCF7L2 interacts with miR-206 in RISCs to inhibit the miR-206-induced invasion of BCa cells. This miR-counteracting effect has also been identified in several long noncoding RNAs [39]. We demonstrated that Cofilin1 is a key pluripotency marker. TCF7L2 forms a RISC with miR-206, binds to the miR-206 promoter region, and suppresses the transcriptional maturation of miR-206, which further targets and upregulates Cofilin1 expression to promote its binding with Cortactin, thereby inducing the formation of invadopodia and the progression of BCa. Nevertheless, this study has several limitations. The significance and association of TCF7L2 and Cofilin1 in the invasion and metastasis of BCa are more complex. To prove this possibility, which is beyond the scope of the current study, several strategies, such as ChIP-seq, immunoprecipitation, and RNA-seq, may be needed, and further studies are needed to obtain insights to better understand these molecules.

In summary, our findings provide a novel perspective for unravelling the mechanisms that determine the invasion and metastasis of BCa at the molecular level. TCF7L2 upregulates Cofilin1 expression to promote the formation of invadopodia in BCa cells, which can affect the binding of Cofilin1 and Cortactin by binding to the miR-206 promoter region and inhibiting the transcriptional maturation of miR-206. In the future, developing a therapy that attenuates the invasiveness of BCa by reducing the expression of Cofilin1 may be possible. Our current study is limited in several respects: while the tail vein injection model validated distal colonization, it does not recapitulate the full metastatic cascade from primary tumor invasion to intravasation. Additionally, the therapeutic potential of targeting the TCF7L2/miR-206 axis remains unverified through *in vivo* pharmacological studies.

## Supporting information

25129Supplementary_Tables

25129supplementary_figures

## Abbreviations

The datasets analyzed in this study are available in the GEO Database ( https://www.ncbi.nlm.nih.gov/geo/), which is openly available for free download.

## Supplementary Data

Supplementary data is available at *Acta Biochimica et Biophysica Sinica* online.
