# Supplementary material for: The TCF7L2/miR-206/Cofilin1 axis promotes the metastasis of bladder cancer cells by regulating the formation of invadopodia: TCF7L2/miR-206/Cofilin1 regulates invadopodia
Source: Acta Biochim Biophys Sin (Shanghai). 2025 Aug 6;58(3):663–80. doi: 10.3724/abbs.2025114 (PMC13059783; doi:10.3724/abbs.2025114)
Supplement: 25129Supplementary_Tables [file 25129Supplementary_Tables.docx]

**Supplementary Table S1. All shRNAs/plasmid used in this study**

| Gene shRNA/plasmid | **Sequence (5′→3′)** | Source |
| --- | --- | --- |
| Cofilin1 shRNA | GCTACGAGGAGGTCAAGGACC | GenePharma Company, Shanghai, China |
| Cofilin1 OE | ATGGCCTCCGGTGTGGCTGTCTCTGATGGTGTCATCAAGGTGTTCAACGACATGAAGGTGCGTAAGTCTTCAACGCCAGAGGAGGTGAAGAAGCGCAAGAAGGCGGTGCTCTTCTGCCTGAGTGAGGACAAGAAGAACATCATCCTGGAGGAGGGCAAGGAGATCCTGGTGGGCGATGTGGGCCAGACTGTCGACGACCCCTACGCCACCTTTGTCAAGATGCTGCCAGATAAGGACTGCCGCTATGCCCTCTATGATGCAACCTATGAGACCAAGGAGAGCAAGAAGGAGGATCTGGTGTTTATCTTCTGGGCCCCCGAGTCTGCGCCCCTTAAGAGCAAAATGATTTATGCCAGCTCCAAGGACGCCATCAAGAAGAAGCTGACAGGGATCAAGCATGAATTGCAAGCAAACTGCTACGAGGAGGTCAAGGACCGCTGCACCCTGGCAGAGAAGCTGGGGGGCAGTGCCGTCATCTCCCTGGAGGGCAAGCCTTTGTGA | GenePharma Company, Shanghai, China |
| TCF7L2 shRNA | TTCCTCCGATTACAGACCTGA | GenePharma Company, Shanghai, China |
| TCF7L2 OE | ATGCCGCAGCTGAACGGCGGTGGAGGGGATGACCTAGGCGCCAACGACGAACTGATTTCCTTCAAAGACGAGGGCGAACAGGAGGAGAAGAGCTCCGAAAACTCCTCGGCAGAGAGGGATTTAGCTGATGTCAAATCGTCTCTAGTCAATGAATCAGAAACGAATCAAAACAGCTCCTCCGATTCCGAGGCGGAAAGACGGCCTCCGCCTCGCTCCGAAAGTTTCCGAGACAAATCCCGGGAAAGTTTGGAAGAAGCGGCCAAGAGGCAAGATGGAGGGCTCTTTAAGGGGCCACCGTATCCCGGCTACCCCTTCATCATGATCCCCGACCTGACGAGCCCCTACCTCCCCAACGGATCGCTCTCGCCCACCGCCCGAACCCTCCATTTTCAGTCCGGCAGCACACATTACTCTGCGTACAAAACGATTGAACACCAGATTGCAGTTCAGTATCTCCAGATGAAATGGCCACTGCTTGATGTCCAGGCAGGGAGCCTCCAGAGTAGACAAGCCCTCAAGGATGCCCGGTCCCCATCACCGGCACACATTGTCTCTAACAAAGTGCCAGTGGTGCAGCACCCTCACCATGTCCACCCCCTCACGCCTCTTATCACGTACAGCAATGAACACTTCACGCCGGGAAACCCACCTCCACACTTACCAGCCGACGTAGACCCCAAAACAGGAATCCCACGGCCTCCGCACCCTCCAGATATATCCCCGTATTACCCACTATCGCCTGGCACCGTAGGACAAATCCCCCATCCGCTAGGATGGTTAGTACCACAGCAAGGTCAACCAGTGTACCCAATCACGACAGGAGGATTCAGACACCCCTACCCCACAGCTCTGACCGTCAATGCTTCCATGTCCAGGTTCCCTCCCCATATGGTCCCACCACATCATACGCTACACACGACGGGCATTCCGCATCCGGCCATAGTCACACCAACAGTCAAACAGGAATCGTCCCAGAGTGATGTCGGCTCACTCCATAGTTCAAAGCATCAGGACTCCAAAAAGGAAGAAGAAAAGAAGAAGCCCCACATAAAGAAACCTCTTAATGCATTCATGTTGTATATGAAGGAAATGAGAGCAAAGGTCGTAGCTGAGTGCACGTTGAAAGAAAGCGCGGCCATCAACCAGATCCTTGGGCGGAGGTGGCATGCACTGTCCAGAGAAGAGCAAGCGAAATACTACGAGCTGGCCCGGAAGGAGCGACAGCTTCATATGCAACTGTACCCCGGCTGGTCCGCGCGGGATAACTATGGAAAGAAGAAGAAGAGGAAAAGGGACAAGCAGCCGGGAGAGACCAATGATGCAAATACTCCAAAGAAGTGTCGGGCACTGTTCGGGCTTGACCGACAGACTTTATGGTGCAAACCGTGCAGGAGAAAAAAAAAGTGCGTTCGCTACATACAAGGTGAAGGCAGCTGCCTCAGCCCACCCTCTTCAGATGGAAGCTTACTAGATTCGCCTCCCCCCTCCCCGAACCTGCTAGGCTCCCCTCCCCGAGACGCCAAGTCACAGACTGAGCAGACCCAGCCTCTGTCGCTGTCCCTGAAGCCCGACCCCCTGGCCCACCTGTCCATGATGCCTCCGCCACCCGCCCTCCTGCTCGCTGAGGCCACCCACAAGGCCTCCGCCCTCTGTCCCAACGGGGCCCTGGACCTGCCCCCAGCCGCTTTGCAGCCTGCCGCCCCCTCCTCATCAATTGCACAGCCGTCGACTTCTTCCTTACATTCCCACAGCTCCCTGGCCGGGACCCAGCCCCAGCCGCTGTCGCTCGTCACCAAGTCTTTAGAATAG | GenePharma Company, Shanghai, China |
| Cortactin shRNA | ACAGAGTTGATCAGTCTGCTG | GenePharma Company, Shanghai, China |
| miR-206 inhibitor | CCACACTUCCTUACAUUCCAUGGAAUGUAAGGAAGUGUGUGGUUUGCCACACUUCCUUACAUUCCA | RiboBio, Guangzhou, China |
| miR-206 mimics | GGAUGGAAUGUAAGGAAGUGUGUGGUUUGUCACACUUCCUUACAUUCCACC | RiboBio, Guangzhou, China |

**Supplementary Table S2. Clinical information of 10 patients with bladder cancer (BCa)**

|  | Gender | BCa  type | WHO  phase | TNM  staging | Grade | Superfical  or invasive |
| --- | --- | --- | --- | --- | --- | --- |
| **1** | Female | Papillary urothelial BCa | Low | T1N0M0 | 1 | Superfical |
| 2 | Male | Invasive BCa | Medium | T1N0M0 | 2 | invasive |
| 3 | Male | Non-invasive BCa | Low | T1N0M0 | 1 | Superfical |
| 4 | Male | Invasive BCa | High | T2N0M0 | 3 | invasive |
| 5 | Male | Invasive BCa | High | T3N0M0 | 3 | invasive |
| 6 | Female | Invasive BCa | High | T3N0M0 | 3 | invasive |
| 7 | Male | Invasive BCa | Medium | T2N0M0 | 2 | invasive |
| 8 | Male | Invasive BCa | High | T3N0M0 | 3 | invasive |
| 9 | Male | Invasive BCa | Medium | T2N0M0 | 2 | invasive |
| 10 | Male | Invasive BCa | High | T3N0M0 | 3 | invasive |

**Supplementary Table S3. Antibodies used in this study**

| Antibodies | Catalog number | Company | Dilution |
| --- | --- | --- | --- |
| For western blot analysis | | | |
| TCF7L2 | ab32072 | Abcam | 1:1000 |
| Cofilin1 | ab42824 | Abcam | 1:1000 |
| GAPDH | sc-365062 | Santa Cruz | 1:1000 |
| Goat Anti-Rabbit IgG | ab136817 | Abcam | 1：20000 |
| For immunohistochemistry/immunofluorescence staining | | | |
| Cofilin1 | ab42824 | Abcam | 1:200 |
| Cortactin | ab81208 | Abcam | 1:1000 |
| Goat anti-rabbit HRP | ab136817 | Abcam | 1:400 |

**Supplementary Table S4. Sequences of primers used in this study**

| Gene | Forward sequence (5′→3′) | Reverse sequence (5′→3′) |
| --- | --- | --- |
| *Cofilin1* | CGCTGCCTCCACAACTACAA | ACACAGGGTGGGTCACAATTT |
| *18srRNA* | CCTGGATACCGCAGCTAGGA | GCGGCGCAATACGAATGCCCC |
| *Homo Cofilin1* | ATAAGGACTGCCGCTATGCC | ACCTCCTCGTAGCAGTTTGC |
| *Homo β-actin* | AGCGAGCATCCCCCAAAGTT | GGGCACGAAGGCTCATCATT |
