# Supplementary material for: The TCF7L2/miR-206/Cofilin1 axis promotes the metastasis of bladder cancer cells by regulating the formation of invadopodia: TCF7L2/miR-206/Cofilin1 regulates invadopodia
Source: Acta Biochim Biophys Sin (Shanghai). 2025 Aug 6;58(3):663–80. doi: 10.3724/abbs.2025114 (PMC13059783; doi:10.3724/abbs.2025114)
Supplement: 25129supplementary_figures [file 25129supplementary_figures.docx]

**
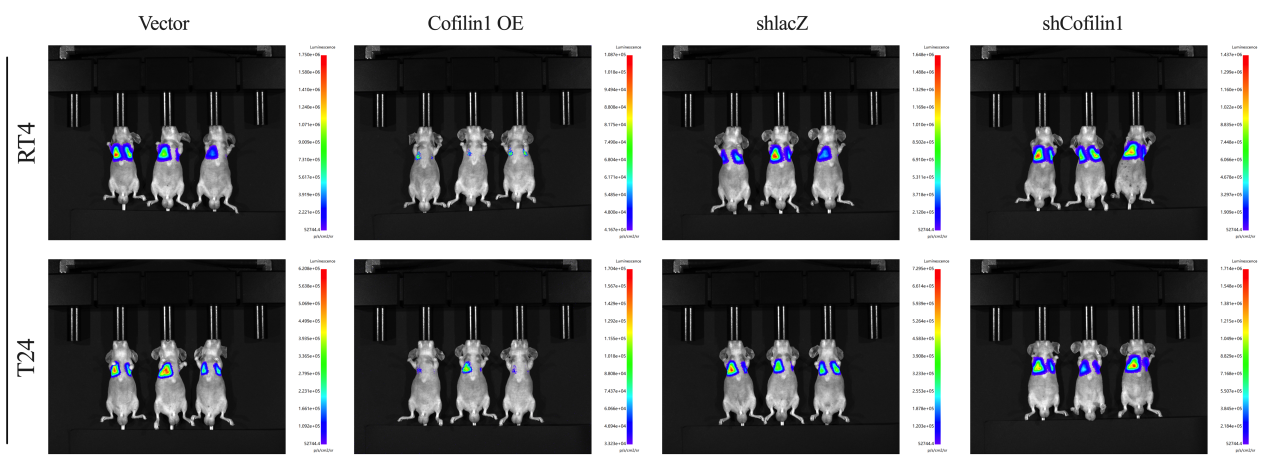
**

**Supplementary Figure S1. Cofilin1 promotes bladder cancer cell metastasis *in vivo***  Tumor metastasis in mice was analyzed using an *in vivo* imaging system after injection of differently treated RT4 and T24 cells through the tail vein of mice.

**
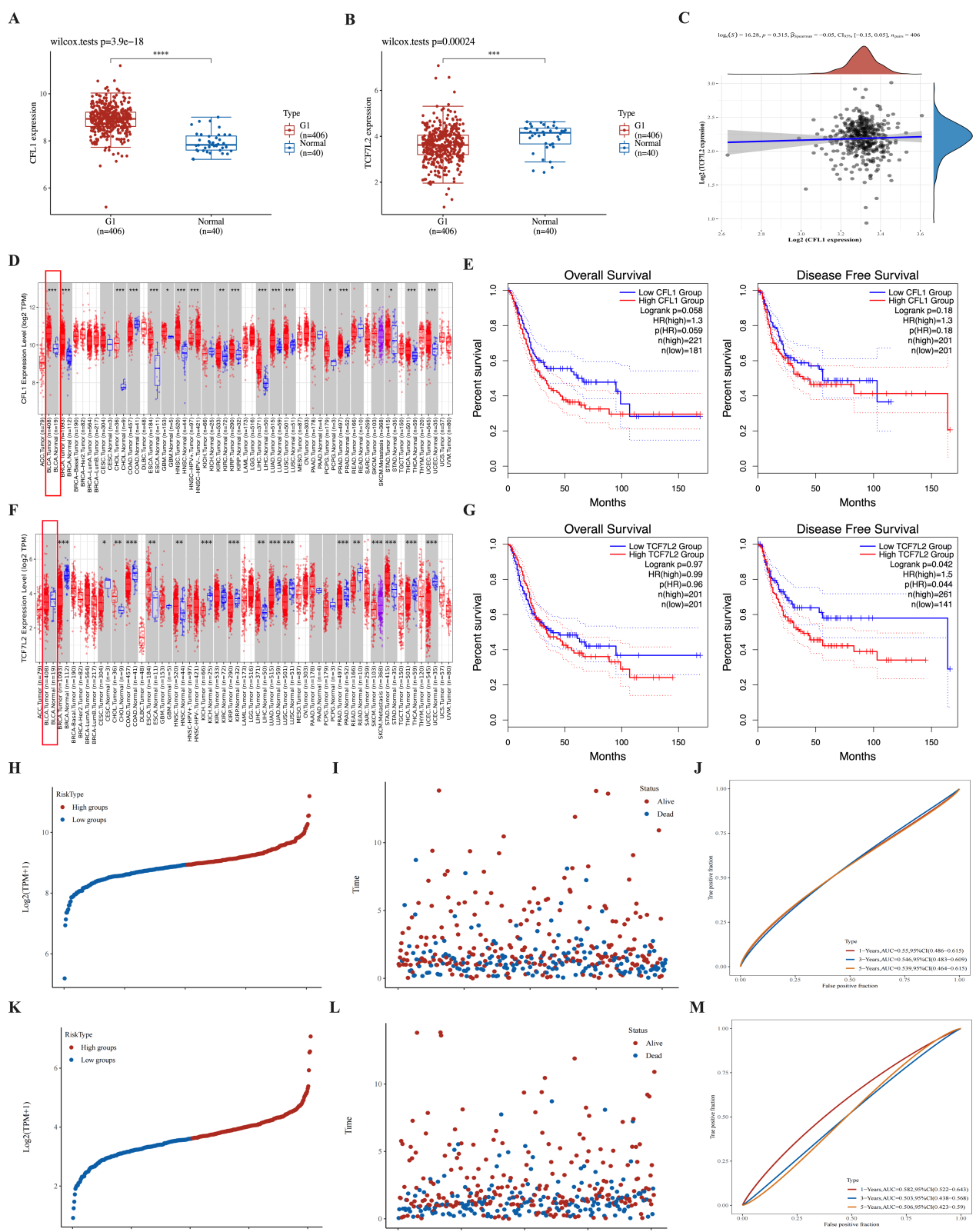
**

**Supplementary Figure S2. Expression levels and clinical correlation analysis of TCF7L2 and Cofilin1 in invasive metastasis of bladder cancer** (A,B) TCGA database analysis of the expression difference between Cofilin1 and TCF7L2 proteins in bladder cancer and normal bladder tissues. ****P* < 0.001, *****P* < 0.0001. (C) Spearman’s correlation analysis between *Cofilin1* and *TCF7L2* gene expressions from the TCGA bladder cancer database. (D,F) TIMER database analysis of *Cofilin1* and *TCF7L2* gene expression levels in pan-cancer. (E,G) Survival prognosis analyses of *TCF7L2* and *Cofilin1* genes using the online analysis tool GEPIA, respectively. (H−J) The TCGA database was used to analyze the relationship between *Cofilin1* gene expression and survival time and survival status. (H) Scatterplot of Cofilin1 expression from low to high, with red representing the high-risk group and blue representing the low-risk group. (I) Scatterplot distribution of patients’ survival time and survival status corresponding to Cofilin1 expression in the high-risk and low-risk groups. (J) Predictive accuracy of the *Cofilin1* gene by TimeROC analysis. (K−M) TCGA database was used to analyze the relationship between *TCF7L2* gene expression and survival time and survival status. (K) Scatterplot of TCF7L2 expression from low to high, with red representing the high-risk group and blue representing the low-risk group. (L) Scatterplot distribution of patients' survival time and survival status corresponding to TCF7L2 expression in the high-risk and low-risk groups. (M) TimeROC analyses Predictive accuracy of the *TCF7L2* gene.

**
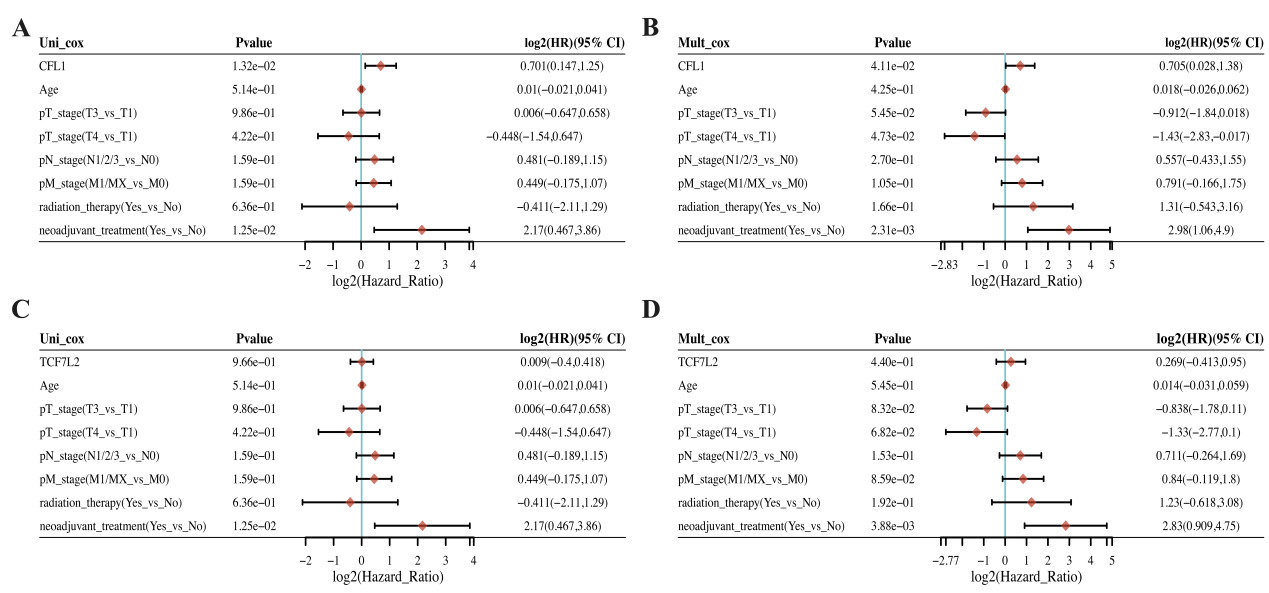
**

**Supplementary Figure S3. Forest plot of multivariate Cox regression analyses for prognostic variables** (A,B) Univariate and multivariate Cox proportional hazards regression analyses showing *P* values, hazard ratios (HR), and 95% confidence intervals (CI) for *Cofilin1* gene expression and clinical characteristics. (C,D) Univariate and multivariate Cox proportional hazards regression analyses of *TCF7L2* gene expression and clinical characteristics, including *P* values, HR, and 95% CI. HR denotes hazard ratio; HR.95%L and HR.95%H represent the lower and upper bounds of the 95% confidence interval, respectively.


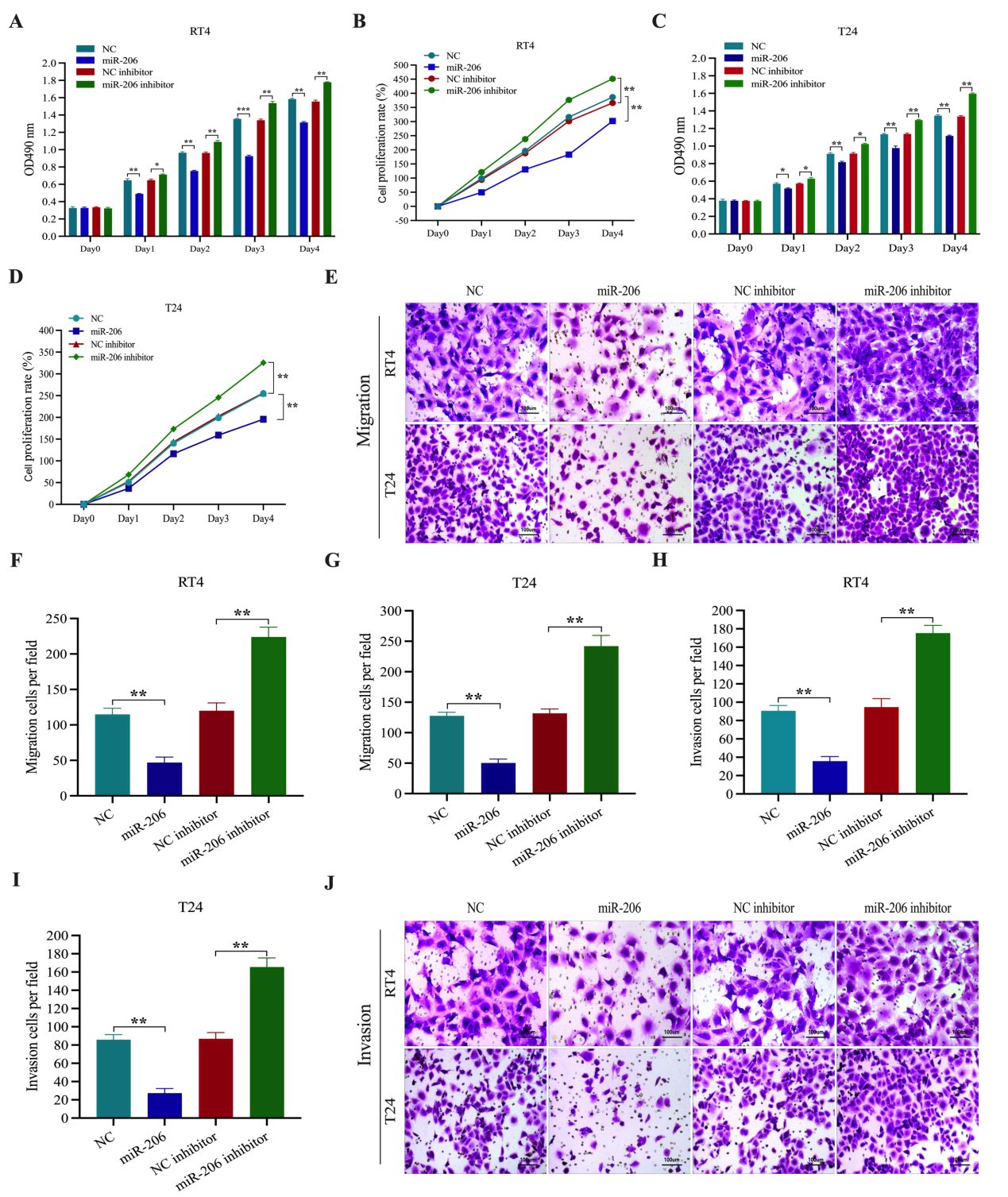


**Supplementary Figure S4. miR-206 inhibits the proliferation, migration and invasion of bladder cancer cells** (A−D) The effects of stable overexpression and inhibition of miR-206 on the proliferation of RT4 and T24 cells were determined (*n* = 3). (E−J) After stable overexpression or inhibition of miR-206, changes in migration (E−G) and invasion (H−J) ability of RT4 and T24 cells were detected by Transwell assay (scale bar = 100 μm). *n* = 3. **P* < 0.05, ***P* < 0.01.
